# Effects of β‐hydroxy β‐methylbutyrate (HMB) supplementation on muscle mass, function, and other outcomes in patients with cancer: a systematic review

**DOI:** 10.1002/jcsm.12952

**Published:** 2022-03-17

**Authors:** Carla M. Prado, Camila E. Orsso, Suzette L. Pereira, Philip J. Atherton, Nicolaas E. P. Deutz

**Affiliations:** ^1^ Human Nutrition Research Unit, Department of Agricultural, Food and Nutritional Science University of Alberta Edmonton AB Canada; ^2^ Research and Development Abbott Nutrition Columbus OH USA; ^3^ Centre of Metabolism, Ageing & Physiology (COMAP), Medical Research Council (MRC) Versus Arthritis Centre for Musculoskeletal Ageing Research (CMAR), and National Institute for Health Research (NIHR) Biomedical Research Centre (BRC) University of Nottingham Nottingham UK; ^4^ Center for Translational Research in Aging and Longevity, Department of Health and Kinesiology Texas A&M University College Station TX USA

**Keywords:** β‐Hydroxy β‐methylbutyrate, HMB, Cancer, Muscle mass, Muscle function, Nutrition intervention

## Abstract

Low muscle mass is prevalent among patients with cancer and a predictor of adverse clinical outcomes. To counteract muscle loss, β‐hydroxy β‐methylbutyrate (HMB) supplementation has been proposed as a potential therapy for older adults and various diseases states. This systematic review aimed to investigate the effects and safety of HMB supplementation in relation to muscle mass and function and other clinical outcomes in patients with cancer. A systematic search of MEDLINE, CINAHL, Embase, Cochrane Central Register of Controlled Trials, Scopus, ProQuest, and grey literature for reports published from inception to December 2021 was conducted. Included studies provided supplements containing any dose of HMB to adult patients with active cancer. A synthesis without meta‐analysis was conducted using a vote‐counting approach based solely on the direction of the effect (i.e. regardless of statistical significance). Risk of bias was assessed for each outcome domain, and evidence from higher‐quality studies (i.e. those with either low or moderate risk of bias) was examined. Safety was evaluated using both lower‐quality and higher‐quality studies. Fifteen studies were included, in which six were randomized controlled trials in patients with various cancer types and treatments. Studies prescribed HMB combined with amino acids (73.3%), HMB in oral nutritional supplements (20.0%), or both supplement types (6.7%); Ca‐HMB doses of 3.0 g/day were provided in 80.0% of the studies. Four studies had high risk of bias across all outcome domains. Considering the higher‐quality studies, evidence of a beneficial effect of HMB supplementation was found in four of four studies for muscle mass, two of two for muscle function, three of three for hospitalization, and five of seven for survival. In contrast, no beneficial effects of HMB on quality of life or body weight was found in two of four and three of five studies, respectively. A limited number of higher‐quality studies evaluating the impact of HMB on cancer therapy‐related toxicity, inflammation, and tumour response were observed. No serious adverse effects directly related to the nutrition intervention were reported. Although limited, current evidence suggests that HMB supplementation has a beneficial effect on muscle mass and function in patients with cancer. Well‐designed trials are needed to further explore the clinical benefit of HMB supplementation in this patient population.

## Introduction

Low muscle mass is prevalent in patients with cancer, and it occurs independent of cancer site, disease stage, and weight loss.^1^, ^2^, ^3^, ^4^, ^5^ Low muscle mass has been consistently associated with adverse clinical outcomes, such as greater mortality, dose‐limiting toxicity, length of hospitalization, and postoperative complications.^2^, ^3^, ^6^, ^7^, ^8^, ^9^ Quality of life is also affected following cancer diagnosis and is positively associated with muscle mass.^10^, ^11^, ^12^, ^13^, ^14^

The pathophysiology of muscle wasting in cancer is multifactorial and not yet fully understood. However, appropriate quality and quantity of nutrients are essential to maintain muscle mass.^15^, ^16^, ^17^, ^18^, ^19^ Several nutrition interventions are under consideration for this purpose, including food supplements (e.g. protein, vitamin D, creatine, and fish oil) and oral nutritional supplements (ONS) administered alone or in combination with nutritional counselling.^15^ One ingredient found in food supplements and specialized, nutrient‐dense ONS with anabolic potential is β‐hydroxy β‐methylbutyrate (HMB).^20^, ^21^ HMB is endogenously produced in the body in small amounts as a product of leucine metabolism—accounting for 0.66% of total leucine turnover.^22^ Although HMB is also present in certain foods (e.g. avocado, catfish, cauliflower, and grapefruit), the level of intake to achieve a therapeutic dose would be impractical (typically 3 g/day administered in experimental and clinical research).^20^, ^21^ This ingredient can be administered as isolated supplement, in combination with amino acids such as arginine and glutamine (HMB/Arg/Gln), or HMB in ONS (HMB‐enriched ONS).^23^

Several pathways have been proposed to explain the effects of HMB on muscle health, which includes both an increase in muscle protein synthesis and a decrease in muscle protein breakdown. HMB stimulates muscle protein synthesis by activating the mechanistic target of the rapamycin (mTOR) system and the growth hormone/insulin‐like growth hormone factor axis.^24^, ^25^, ^26^ HMB is also associated with a reduction in muscle proteolysis and apoptosis of myonuclei by inhibiting ubiquitin–proteasome and the autophagy–lysosome systems, respectively.^27^, ^28^, ^29^ The benefits of HMB on muscle health (i.e. mass and function) have been widely explored in preclinical and clinical studies including older adults and different disease conditions.^20^, ^21^ For example, findings of a 2019 meta‐analysis including 15 randomized controlled trials (RCTs) in patients with different clinical conditions (including two in cancer) have shown an overall positive effect (although small) on muscle mass and strength of HMB at the most tested dose of 3.0 g/day.^23^

HMB's anabolic potential is being exploited to address muscle loss in cancer, with potential to improve long‐term outcomes. Yet as reported in oncology guidelines, the lack of consistent clinical evidence is still a challenge for recommending supplements containing branched‐chain amino acids and their metabolites such as HMB.^30^, ^31^ Thus, our aim was to conduct a comprehensive review of existing evidence on the effects of HMB supplementation on muscle mass and function and other clinical outcomes in patients with active cancer. We also assessed the evidence of HMB safety in this population.

## Methods

This systematic review was conducted in accordance with the Preferred Reporting Items for Systematic Review and Meta‐analysis (PRISMA)^32^ as well as the Synthesis Without Meta‐analysis (SWiM)^33^ reporting guidelines. The review protocol was registered with PROSPERO (CRD42021273890).

### Search strategy and selection criteria

The search strategy was developed combining key terms and medical subject headings relating HMB supplements and oncology patients (*Supporting Information* *S1*). Searches were carried out in MEDLINE (via Ovid), CINAHL, Embase (via Elsevier), Cochrane Central Register of Controlled Trials (CENTRAL), Scopus, and ProQuest Dissertations & Theses Global from inception to 12 December 2021 (last search date). Restrictions for English language and human studies were used. Records identified through database searches were imported to Covidence software (Veritas Health Innovation Ltd, Melbourne, Victoria, Australia) for automatic deduplication and study selection by two independent reviewers. Titles, abstracts, and full texts were reviewed against eligibility criteria, as detailed in *Supporting Information* *S2*. Briefly, we included RCTs and non‐randomized studies of interventions (NRSIs) exploring the effects of HMB supplementation (of any form and dose) on muscle mass and several other health and safety outcomes in adult patients with active cancer. Studies with mixed populations (i.e. patients with cancer and patients with other diseases) were included only if outcome data was stratified by patient population. Conference abstracts, case reports, articles not including original and peer‐reviewed data, and studies terminated earlier than planned were excluded. Disagreement were resolved by discussion or a third reviewer, if necessary. Additional searches were conducted by one reviewer in ClinicalTrials.gov and Google (last search date 12 December 2021), and the reference lists of retrieved reports and relevant reviews were manually searched for potentially eligible titles.

### Outcomes

Our primary outcomes included muscle mass and related terms [i.e. fat‐free mass (FFM), lean soft tissue, skeletal muscle, and anthropometric surrogates] and health‐related quality of life. Additional outcomes were body weight [or body mass index (BMI)], muscle function (i.e. muscle strength and physical performance), prevalence of muscle‐related abnormalities (i.e. low muscle mass or function, sarcopenia, or cachexia), inflammatory markers, cancer therapy‐related toxicities, hospitalization rate, length of hospital stay, postoperative complications, tumour response, mortality and survival, and safety. Further details are included in the eligibility criteria (*Supporting Information* *S2*).

### Data extraction

A single reviewer extracted data on the characteristics of included reports, and a second reviewer checked the collected information for accuracy using an online spreadsheet (Google Sheets). Disagreements were discussed until a consensus was reached. General study information was extracted (i.e. first author, publication date, country, and aims) as well as study details (i.e. design, participants, interventions, statistical approaches, outcome assessment, and results) and miscellaneous (e.g. overall conclusions, limitations, and potential conflict of interest).

For each outcome, we collected the following information, when available: measurement tools, person performing the assessment, measured and reported time points, unit of measure, number of participants with valid measure, baseline and follow‐up data (i.e. time point closest to the end of the nutrition intervention) for experimental and control groups, effect size and corresponding *P*‐value, and any reported subgroup analysis. Measures of effect size included change from baseline to follow‐up within the experimental arm [for both controlled (i.e. with intervention and control arms) and uncontrolled (i.e. with an intervention arm alone) studies], differences in change between the experimental and control arms (for controlled studies only when data were available), and differences between experimental and control arms at follow‐up (for outcomes related to cancer therapy‐related toxicities, hospitalization, survival, and tumour response in controlled studies).

Data available only in figures were extracted using the Plot Digitizer software (V.2.6.9; http://plotdigitizer.sourceforge.net).^34^ When a specific study had findings published in multiple articles, unique data were extracted, and these reports were treated as a single study. Studies with mixed populations had outcome data extracted only when pertaining to those with cancer. We also collected data on adherence to the intervention protocol and measures of total energy and protein intakes, when available. If necessary, corresponding authors were contacted for additional information, and websites of nutritional supplement were accessed for supplement composition. The included studies used products containing 1.5 g of Ca‐HMB per serving of the supplement, which is equivalent to 1.2 g of HMB. For consistency across the studies, we have selected to report the dose as that of Ca‐HMB (1.5 g per serving; if two servings were administered, patients received a total of 3.0 of Ca‐HMB per day).

### Risk‐of‐bias assessment

All included studies were independently rated by two reviewers using either the Revised Cochrane risk‐of‐bias tool for randomized trials (RoB2)^35^ or the Risk Of Bias In Non‐Randomized Studies (ROBINS‐I)^36^ to evaluate the quality of RCTs and NRSIs, respectively. A separate risk‐of‐bias assessment was completed for each study and outcome domain. Results were graphically summarized using the Risk‐of‐bias VISualization (robvis) tool.^37^

### Data synthesis

Findings are organized according to outcome domains, study design (i.e. RCT or NRSI), and HMB supplement type (i.e. HMB/Arg/Gln or HMB‐enriched ONS). Related outcomes were grouped into outcome domains: quality of life, cancer‐related fatigue, and symptom assessment were summarized into the ‘quality of life domain’; muscle strength and physical performance were combined into the ‘muscle function’ domain; and readmission rate, length of stay, and postoperative complications were evaluated into the ‘hospitalization domain’.

Because of variation in the methods used to assess and report outcomes across studies, we synthesized the evidence using a vote‐counting approach based solely on the direction of effect (i.e. regardless of statistical significance or testing), as previously recommended.^33^, ^38^ When available data permitted, direction of effect was defined for each outcome domain and type of effect size measure as ‘beneficial effect’, ‘mixed effect’, or ‘no beneficial effect’. A ‘beneficial effect’ was reported if the intervention had a positive health impact or the outcome remained unchanged, with the latter indicating that patients receiving HMB maintained the outcome of interest. In contrast, HMB supplement was described as having ‘no beneficial effect’ if the desired benefit of the intervention was not obtained and/or there was a worsening of the outcome. For outcome domains summarizing multiple related outcomes, HMB supplement was classified as having either ‘beneficial effect’ or ‘no beneficial effect’ if ≥70% of outcomes reported similar direction; studies were labelled as ‘mixed effect’ if <70% of outcomes had consistent direction of effect.^39^

Findings from each individual study were graphically presented using both the Harvest plot and the effect of direction plot (provided as *Figure* *S1*), which also allowed us to synthesize the evidence based on the study size and risk of bias.^39^, ^40^, ^41^ Given the challenges to perform research in clinical nutrition and cancer as deterioration of patients' condition can be related to response to cancer therapy,^42^, ^43^ we defined studies with final sample size (i.e. number of patients) in the experimental arm <20 as small, 20–50 as medium, and >50 as large studies. We also graphically tabulated the available effect estimates and *P*‐values for each individual study. We calculated the mean or median differences between baseline and follow‐up within study arms when absolute or percent change values were not reported. To draw conclusions on whether there was an effect of HMB supplementation on muscle mass, function, quality of life, and clinical outcomes, a sensitivity analysis was conducted in which we evaluated findings from studies with low and moderate risk of bias combined.^36^ Direction of effect was summarized based on the difference in changes between groups (or follow‐up differences) in controlled studies and changes from baseline to follow‐up within the experimental arm in uncontrolled studies. We were unable to assess studies with a low risk of bias alone because of their limited number.

## Results

**Figure 1:**
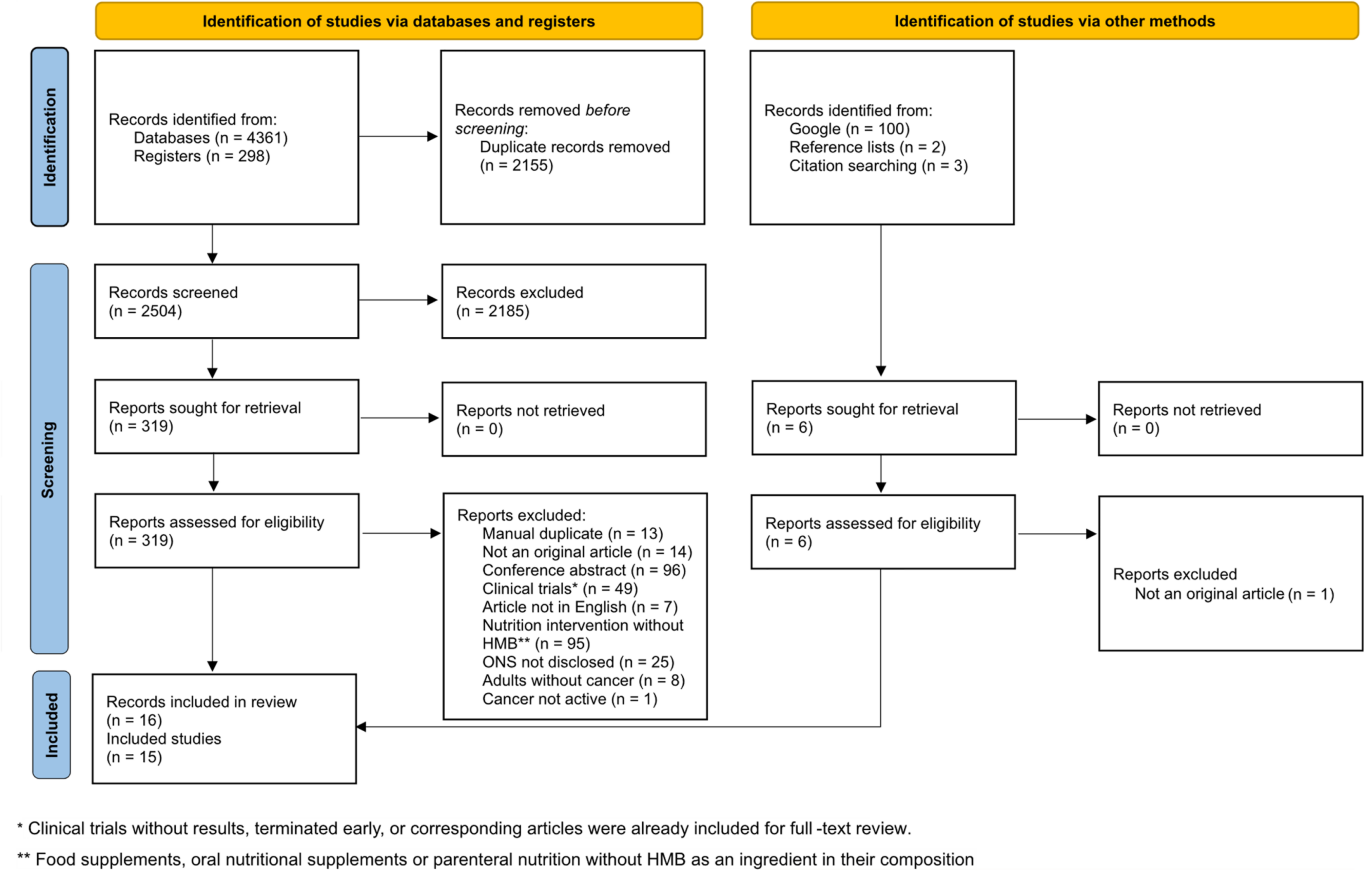
PRISMA 2020 flow diagram for study selection.

A total of 4764 records were identified in databases, registries, and other sources (*Figure* 1). After removing duplicates and screening titles/abstracts, we reviewed 325 full‐text records. Of those, 16 reports published between 2002 and 2021 were deemed eligible for inclusion, with two reports describing different outcomes from the same study.^44^, ^45^ Therefore, 15 unique studies involving 943 individuals were included. Studies were conducted in Japan,^46^, ^47^, ^48^, ^49^, ^50^ Turkey,^51^, ^52^, ^53^, ^54^ the USA,^44^, ^55^, ^56^ Spain,^57^, ^58^ and Italy.^59^

### Study characteristics

*Table* 1 displays detailed characteristics of studies stratified by design. There were six RCTs (40% of all studies), including a total of 416 patients (mean or median age range of 62.0–69.0 years) undergoing treatment for cancers of diverse types and stages. Five RCTs administered HMB/Arg/Gln,^44^, ^46^, ^47^, ^54^, ^55^ and an HMB‐enriched ONS was provided to patients in one RCT.^56^ Supplement was orally administered in most studies (four of five), except for two using tube feeding or percutaneous endoscopic gastrostomy.^46^, ^54^ Length of intervention ranged from 10 days (3 days preoperatively plus 7 days postoperatively) to 8 weeks, with one study being conducted over 6 months.^44^ None of the RCTs employed multimodal interventions.

**Table 1:** Study characteristics

Reference	Study design	*N* (*n* Int, *n* Ctr)	Population characteristics	Cancer therapy	Intervention	Control	Length of intervention	Adherence
May, 2002, and Rathmacher, 2004	RCT (double‐blind, multicentre)	32 (18 Int, 14 Ctr)	Advanced solid tumours; Stage IV; >5% weight loss Age, mean ± SD: 64.3 ± 2.4 Int, 67.4 ± 2.4 Ctr	Chemotherapy, radiotherapy	HMB/Arg/Gln 2 × Ca‐HMB 1.5 g, l‐arginine 7 g, and l‐glutamine 7 g doses/day	Isonitrogenous, isocaloric placebo 2 × l‐alanine 5.5 g, l‐glutamic acid 0.88 g, l‐glycine 3.05 g, and l‐serine 2.11 g doses/day	24 weeks	Not reported

Berk, 2008	RCT (double‐blind, multicentre)	197 (106 Int, 91 Ctr)	Stage III–IV solid cancer, or currently metastatic cancer of any initial stage; 2–10% weight loss over the previous 3 months Age, median (range): 65 (34–91)	Chemotherapy (*n* = 100)	HMB/Arg/Gln 2 × Ca‐HMB 1.5 g, l‐arginine 7 g, and l‐glutamine 7 g doses/day	Isonitrogenous, isocaloric placebo 2 × l‐alanine 3.86 g, l‐glutamic acid 0.62 g, l‐glycine 2.14 g, l‐serine 1.48 g, and gelatin 15.3 g doses/day	8 weeks	Trial was completed per protocol by 88 patients in Int vs. 77 patients in Ctr group

Imai, 2014	RCT (open‐label, single‐centre)	34 (16 Int, 18 Ctr)	Head and neck cancer; Stages II–IV Age, mean (range): 62 (42–74) Int, 64 (41–76) Ctr	CCRT	HMB/Arg/Gln 2 × Ca‐HMB 1.5 g, l‐arginine 7 g, and l‐glutamine 7 g doses/day + Routine nutritional care (prophylactic enteral tube feeding)	Standard care + Routine nutritional care (prophylactic enteral tube feeding)	From the first day of CCRT up to until 1 week after the completion of CCRT	Supplement was taken by 15 patients in Int until the end of intervention

Yildiz, 2016	RCT [single‐blind (randomization team), single‐centre]	41 (21 Int, 20 Ctr)	Cancers of the distal oesophagus, stomach, and head of the pancreas; Stage ≤ III Age, mean ± SD: 64.05 ± 9.04 Int, 62.60 ± 9.10 Ctr	Surgery	HMB/Arg/Gln 2 × Ca‐HMB 1.5 g, l‐arginine 7 g, and l‐glutamine 7 g doses/day + High‐protein ONS + Routine nutritional care (postoperative nutritional protocol)	High‐protein ONS + Routine nutritional care (postoperative nutritional protocol)	7 days preoperatively + 7 days postoperatively	Not reported


Wada, 2018	RCT (double‐blind, single‐centre)	60 (30 Int, 30 Ctr)	Gastrointestinal cancers Age, median (range): 66 (40–81) Int, 69 (25–81) Ctr	Surgery	HMB/Arg/Gln 1 × Ca‐HMB 1.5 g, l‐arginine 7 g, and l‐glutamine 7 g doses/day + Routine nutritional care (regular hospital diet)	Isocaloric placebo + Routine nutritional care (regular hospital diet)	3 days preoperatively + 7 days postoperatively	Patients consumed 95% and 90% of the planned volume in Int and Crt, respectively

Ritch, 2019	RCT (open‐label, single‐centre)	52 (28 Int, 24 Ctr)	Bladder cancer; cT1–cT4, N0, M0 Age, median: 69 Int, 67 Ctr	Surgery; neoadjuvant chemotherapy in 58% Int and 47% Ctr	HMB‐enriched ONS 2 × Ca‐HMB 1.5 g doses/day + Routine nutritional care (standard clinical care pathway postoperatively)	Multivitamin + Routine nutritional care (standard clinical care pathway postoperatively)	3–4 weeks preoperatively + 4 weeks postoperatively	Compliance rate was 88% and 85% in Int and Crt, respectively

Previtali, 2020	Non‐randomized trial (open‐label, single‐centre)	35 (19 Arm 1, 13 Arm 2, 3 Arm 3)	Retroperitoneal soft tissue sarcoma; Grades 1–3; patients in Arms 2 and 3 had protein energetic malnutrition and >5% weight loss over the previous 6 months Age, median (range): 59 (34–74)	Neoadjuvant radiotherapy (*n* = 8); neoadjuvant chemotherapy (*n* = 7); chemotherapy/radiotherapy (*n* = 1); surgery only (*n* = 19)	Arm 1: HMB‐enriched ONS 1 × Ca‐HMB 1.5 g doses/day Arm 2: HMB‐enriched ONS + HMB/Arg/Gln 2 × Ca‐HMB 1.5 g doses/day Arm 3: HMB‐enriched ONS + HMB/Arg/Gln 4 × Ca‐HMB 1.5 g doses/day + Routine nutritional care (enteral or parenteral nutrition in order to reach caloric target when needed)	Not applicable	Arm 1: 5 days preoperatively; Arms 2 and 3: 15 days preoperatively	Adherence to preoperative HMB‐enriched ONS was 91% in the overall group

Yuce Sari, 2016	Controlled before‐and‐after study	29 (15 Int, 14 Ctr)	Head and neck cancer; early stage and locally advanced stage Age, median (range): 60 (32–80) Int, 55 (34–66) Ctr	IMRT	HMB/Arg/Gln 2 × Ca‐HMB 1.5 g, l‐arginine 7 g, and l‐glutamine 7 g doses/day	Standard care	From the first day of IMRT up to until the last day of IMRT	Not reported

Naganuma, 2019	Historically controlled study (open‐label, single‐centre)	50 (25 Int, 25 Ctr)	Hepatocellular carcinoma; Stages II–IV Age, mean ± SD: 72.4 ± 12.1 Int, 68.2 ± 7.6 Ctr	Sorafenib	HMB/Arg/Gln 1 × Ca‐HMB 1.5 g, l‐arginine 7 g, and l‐glutamine 7 g doses/day + Routine nutritional care (nutritional counselling)	Standard care + Routine nutritional care (nutritional counselling)	12 weeks	24 patients in Int consumed the supplement per protocol; one patient consumed half of one serving

Yamamoto, 2017	Single‐arm trial	22	Gastric cancer; Stages I–IV; sarcopenia (EWGSOP1 definition) Age, mean: 75	Surgery	HMB/Arg/Gln^a^ 2 × Ca‐HMB 1.5 g, l‐arginine 7 g, and l‐glutamine 7 g doses/day + Routine nutritional care (nutritional counselling) + Exercise programme	Not applicable	16 days (range 7–26 days) preoperatively	Completion rate was 54.5%

Yokota, 2018	Single‐arm trial	35	Head and neck cancer Age, median (range): 62 (20–73)	CCRT	HMB/Arg/Gln 2 × Ca‐HMB 1.5 g, l‐arginine 7 g, and l‐glutamine 7 g doses/day + Routine nutritional care (percutaneous endoscopic gastrostomy for patients unable to eat adequately or hydrate orally)	Not applicable	First day until the last day of radiotherapy	Of the 29 patients with adherence data available, mean compliance was 82.7% (11.8–100%)

Saka, 2019	Prospective cohort study	55	Cancer type and stage were not reported; all patients had malnutrition according to SGA Age^b^, mean ± SD: 74.7 ± 6.8	Not reported	HMB/Arg/Gln 2 × Ca‐HMB 1.5 g, l‐arginine 7 g, and l‐glutamine 7 g doses/day + Routine nutritional care (energy and protein‐rich diet and/or enteral nutrition if insufficient oral intake)	Not applicable	36 days	58.2% of patients were still consuming two servings of supplement at Visit 2

de Luis, 2018	Prospective cohort study (multicentre)	60	Cancer type and stage were not reported; all patients were at risk of malnutrition; 92.4%^b^ patients had weight loss in the previous 3 months Age^b^, mean ± SD: 80.7 ± 8.24	Not reported	HMB‐enriched ONS 2 × Ca‐HMB 1.5 g doses/day	Not applicable	12 weeks	Not reported

Parlak, 2020	Retrospective cohort study (single‐centre)	86 (19 Int, 48 Ctr1, 19 Ctr2)	Cancer of any type and stage; none of the patients had metastases; all patients had malnutrition according to NRS 2002 Age, mean ± SD: 69.00 ± 4.38 Int, 66.50 ± 4.55 Ctr1, 68.21 ± 5.74 Ctr2	Chemotherapy: *n* = 39; radiotherapy: *n* = 4; concurrent chemoradiotherapy: *n* = 7; none: *n* = 36; surgery: *n* = 40	HMB/Arg/Gln 2 × Ca‐HMB 1.5 g, l‐arginine 7 g, and l‐glutamine 7 g doses/day + Routine nutritional care (hospital food and enteral nutrition to reach target caloric intake)	Ctr1: Gln 6 × 5 g l‐glutamine Ctr2: High‐protein ONS + Routine nutritional care (hospital food and enteral nutrition to reach target caloric intake)	Mean ± SD: 10.42 ± 5.73 days	Not reported

Cornejo‐Pareja, 2021	Retrospective cohort study (single‐centre)	155	Cancer of any type; patients were malnourished (75.5%) or at risk of malnutrition (24.5%) according to SGA	Chemotherapy, radiotherapy	HMB‐enriched ONS 2 × Ca‐HMB 1.5 g doses/day + Routine nutritional care (nutritional counselling) + Exercise recommendations	Not applicable	3–6 months	Not reported for oncology cohort

Arg, arginine; CCRT, concurrent chemoradiotherapy; Ctr, control group; EWGSOP1, European Working Group on Sarcopenia in Older People; Gln, glutamine; HMB, β‐hydroxy β‐methylbutyrate; IMRT, intensity‐modulated radiotherapy; Int, intervention group; NRS, Nutrition Risk Screening; ONS, oral nutritional supplements; RCT, randomized controlled trial; SD, standard deviation; SGA, subjective global assessment.

^a^ A Japanese study reported the supplement as having a 2.4 g of HMB dose per day but did not mention whether it was administered in combination with Arg and Gln. Corresponding authors were contacted to clarify, but no response was obtained. As Japan has strict laws about dietary supplements and the Abound product (by Abbott™) is a registered drug in the country, we described it here as an HMB/Arg/Gln supplement.

^b^ Study with mixed population; reported data correspond to the overall study population (not specific to patients with cancer).

Nine NRSIs (60.0%) of varied designs met the eligibility criteria, including a total of 527 patients (mean or median range: 62.3–69.0 years) with diverse cancer types and stages. A supplement containing HMB/Arg/Gln was taken by patients in seven studies.^48^, ^49^, ^50^, ^51^, ^52^, ^53^, ^59^ Of these, one trial^59^ prescribed HMB/Arg/Gln in combination with HMB‐enriched ONS for patients with mild or severe protein‐energy malnutrition. The effects of supplementation with HMB‐enriched ONS alone were also evaluated in two cohort studies.^57^, ^58^ Most studies administered HMB supplements orally^49^, ^50^, ^51^, ^52^, ^53^, ^57^, ^58^ and two via tube feeding.^49^, ^53^ A multimodal intervention composed of nutrition plus exercise programme was administered in two studies.^50^, ^58^

### Primary outcomes

#### Muscle mass

**Figure 2:**
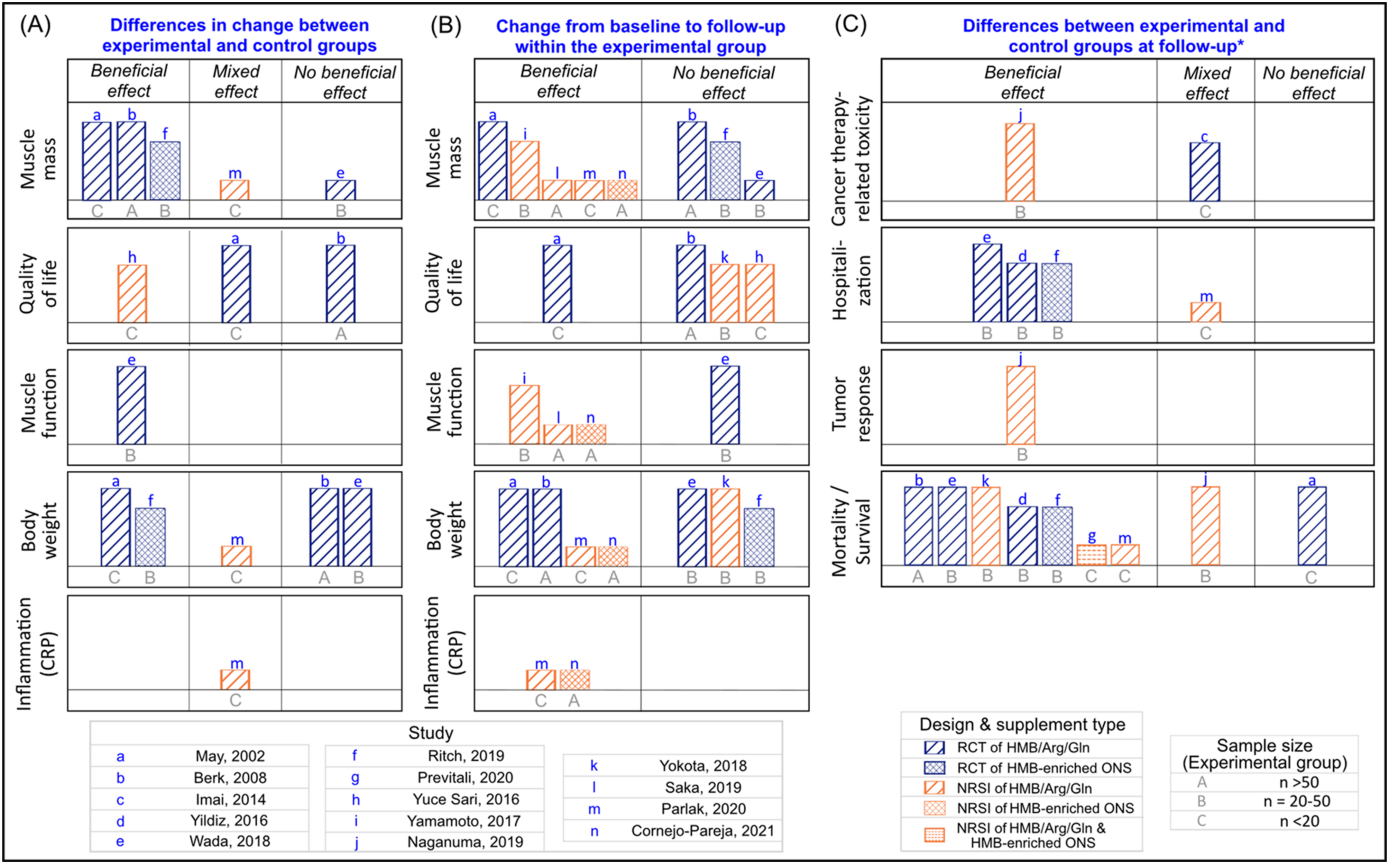
Harvest plots summarizing the effects of β‐hydroxy β‐methylbutyrate (HMB) on muscle mass, function, quality of life, and other outcomes in patients with cancer. (A) Describes the effects based on the differences in change of outcomes between experimental and control groups. (B) Indicates the effects within experimental arm considering changes from baseline to follow‐up; no studies reported mixed effects. (C) Represents direction of effects based on the differences between experimental and control groups at follow‐up, *except for uncontrolled studies reporting on mortality rate in which the number of deaths within the group was considered (i.e. a beneficial effect if no deaths occurred). The height of the bar describes the study quality, with taller bars indicating low risk of bias, mid‐height bars representing moderate risk of bias, and shorter bars illustrating high risk of bias. Each lowercase letter represents a distinct study presented in the figure, and uppercase letters indicate sample size of experimental groups. Study design and supplement type are depicted by different colour and hatch patterns, respectively. As an example of interpretation, the RCT by Berk *et al*. administered HMB/Arg/Gln to more than 50 patients in the experimental arm; the study found that supplements containing HMB had no beneficial effect on muscle mass within the experimental arm alone, but a beneficial effect was observed when results were compared between the experimental and control groups. Arg, arginine; CRP, C‐reactive protein; Gln, glutamine; ONS, oral nutritional supplement.

Four RCTs^44^, ^47^, ^55^, ^56^ and four NRSIs^50^, ^51^, ^53^, ^58^ reported measures of muscle mass (*Figures* 2 and S2). Of these, six estimated FFM using either hand‐to‐foot^44^, ^55^ or foot‐to‐foot^47^, ^58^ single‐frequency bioelectrical impedance analysis (BIA) or a multi‐frequency BIA.^50^, ^51^ Furthermore, one RCT^56^ assessed skeletal muscle by computed tomography, and three NRSIs^51^, ^53^, ^58^ evaluated anthropometric measures. Two RCTs also measured FFM by air‐displacement plethysmography^44^, ^55^ in a small subset of patients, and therefore, findings were not included in the synthesis.

A beneficial effect of HMB supplementation on muscle mass was found in three of four RCTs^44^, ^55^, ^56^ compared with the control group; five studies (one RCT^44^ and four NRSIs^50^, ^51^, ^53^, ^58^) found a beneficial effect within the experimental arm alone. Furthermore, prevalence of low muscle mass did not change in patients receiving an HMB‐enriched ONS in one RCT,^56^ although there was an increase in the incidence of low muscle mass in the control group (between groups: *P* = 0.01). HMB supplementation was additionally associated with a lower prevalence of sarcopenia at follow‐up compared with baseline in one NRSI.^50^

Overall, most RCTs showed a beneficial effect of HMB supplementation on muscle mass compared with controls but not within the experimental arm alone in patients with cancer. In contrast, all NRSIs showed a beneficial effect of HMB from baseline to follow‐up in patients consuming HMB supplements.

#### Quality of life

Quality of life was evaluated in two RCTs^44^, ^55^ and two NRSIs^49^, ^52^ using different assessment tools (*Figure* S3). Compared with controls, supplementation with HMB/Arg/Gln had a beneficial effect on quality of life in one NRSI,^52^ mixed effects in one RCT,^44^ and no beneficial effects in another RCT^55^ (*Figures* 2 and S3). From baseline to follow‐up, patients consuming HMB containing supplements improved their quality of life in one RCT,^44^ but no improvement was shown in three other studies.^49^, ^52^, ^55^ Altogether, the findings indicate a heterogeneous effect of HMB supplementation on quality of life in patients with cancer.

### Additional outcomes

#### Muscle function

Measures of handgrip strength were reported in one RCT^47^ and three NRSIs,^50^, ^51^, ^58^ and the 4 m gait speed test was used in one NRSI^50^ (*Figures* 2 and S4). Although the RCT showed no beneficial effect of the intervention in the experimental arm alone, patients who received the HMB/Arg/Gln supplement had a smaller decrease in handgrip strength compared with controls.^47^ All NRSIs reported a beneficial effect of HMB supplementation on increasing muscle function at follow‐up.^50^, ^51^, ^58^ In summary, HMB showed some effects on muscle function in RCT and NRSIs.

#### Body weight

Compared with controls (*Figures* 2 and S5), a beneficial effect of HMB was found in two RCTs^44^, ^56^ and mixed effects in one NRSI.^53^ HMB supplements had a beneficial effect on increasing body weight (or BMI) with a dose of 3 g/day of Ca‐HMB over 4–24 weeks in two RCTs^44^, ^55^ and one NRSI,^58^ but no beneficial effect when a dose of 1.5–3.0 g/day of Ca‐HMB was administered in the perioperative period in two RCTs.^47^, ^56^ BMI was maintained at follow‐up in a prospective cohort providing patients with HMB/Arg/Gln for a mean of 10.42 (SD 5.73) days during hospital stay.^53^ Conversely, a single‐arm trial reported no beneficial effects of HMB/Arg/Gln, with 17.1% of patients experiencing 10–20% weight loss at follow‐up.^49^ Overall, the effect of HMB supplementation on body weight was heterogeneous.

#### Cancer therapy‐related toxicity

Cancer therapy‐related toxicity was evaluated in one RCT^46^ and three NRSIs^48^, ^49^, ^52^ using the Common Terminology Criteria for Adverse Events (Versions 3.0 and 4.0) and the Radiation Therapy Oncology Group Cooperative Group Common Toxicity Criteria (*Figures* 2 and S6). Compared with controls at follow‐up, supplementation with HMB/Arg/Gln had mixed effects on the incidence of dermatitis in the RCT^46^ but beneficial effects on sorafenib‐associated toxicities in one historically controlled study.^48^ We could not synthesize findings from two NRSIs as only *P*‐values were reported^52^ or no control group was available.^49^ In these studies, a range of cancer therapy‐related toxicities were recorded including oral mucositis, stomatitis, oral pain, and dysphagia. In summary, in spite of the limited number of studies, HMB supplementation showed some beneficial effects on cancer therapy‐related toxicity.

#### Hospitalization

Three RCTs^47^, ^54^, ^56^ and three NRSIs^50^, ^53^, ^59^ in patients undergoing surgery reported data on hospitalization outcomes, including 30 day readmission rate, length of stay, and postoperative complications (*Figures* 2 and S7). Compared with controls, the RCTs^47^, ^54^, ^56^ showed a beneficial effect on these outcomes with either preoperative or postoperative HMB supplementation. Of the two NRSIs reporting on preoperative nutrition interventions, Parlak and Atalay^53^ showed mixed effects of HMB/Arg/Gln supplementation: length of stay was longer in the experimental arm compared with patients receiving glutamine alone, but shorter compared with patients receiving ONS without HMB. Furthermore, it was not possible to synthesize findings from Previtali *et al*.^59^ and Yamamoto *et al*.^50^ as data were presented for all patients vs. stratified by the different intervention administered to patients in the former, or no control group was available in the latter. Altogether, these four studies indicate some beneficial effects of HMB containing supplements on hospitalization outcomes in patients with cancer.

#### Inflammation and tumour response

A beneficial effect of HMB supplementation on reducing serum C‐reactive protein levels was shown in two NRSIs^53^, ^58^ (*Figure* 2); however, controls receiving glutamine alone presented with greater reductions in this inflammatory marker.^53^ Supplementation with HMB/Arg/Gln also had a beneficial effect on tumour response in patients undergoing sorafenib treatment in another NRSI.^48^ Compared with historical controls, tumour response rate was similar and a greater disease control rate was found in patients receiving the supplement^48^ (*Figure* 2). Overall, there were a limited number of studies showing beneficial effects of HMB supplements on serum C‐reactive protein and tumour response.

#### Mortality and survival

Five RCTs^44^, ^47^, ^54^, ^55^, ^56^ and four NRSIs^48^, ^49^, ^53^, ^59^ reported data on mortality, which was recorded from the start to the end of the intervention or to a follow‐up period ranging from 30 days to 7 years (*Figures* 2 and S8). Only one study distinguished between cancer‐specific and all‐cause mortality,^56^ and another reported the cause of death.^48^ Compared with controls, a beneficial effect of HMB supplements on lower mortality rate during the intervention period and at a 30 day follow‐up was found in four of five RCTs.^47^, ^54^, ^55^, ^56^ Three NRSIs also showed a beneficial effect of the experimental treatment on reducing mortality, reporting no deaths during HMB supplementation or after surgery,^49^, ^59^ and a lower hospital mortality as well as 2 year mortality rate.^53^ Mixed effects were found in one NRSI,^48^ with patients in the experimental arm presenting with a greater survival time and rate at 1 year than historical controls but lower at the 2 year follow‐up period. The combined findings of RCTs and NRSI suggest a beneficial effect of HMB supplementation on measures of mortality or survival.

### Safety

Absolute number or percentage of adverse events were described in three RCTs^44^, ^46^, ^55^ and seven NRSIs^48^, ^49^, ^50^, ^51^, ^53^, ^57^, ^59^ (*Supporting Information* *S3*). Incidence of gastrointestinal adverse events was ≤15%, with two studies reporting a probable relationship between these events and HMB/Arg/Gln supplementation.^51^, ^55^ No serious adverse events related to the nutrition intervention were recorded in three studies.^51^, ^57^, ^59^ Despite not separating data for each clinical condition, the safety data from de Luis et al.^57^ was included in this analysis as no patients presented with serious adverse events. Importantly, supplementing the diet with 3.0 g/day of Ca‐HMB was safe and well tolerated for over 24 weeks.^44^

Four RCTs^45^, ^47^, ^54^, ^56^ and four NRSIs^49^, ^51^, ^53^, ^58^ reported data on blood chemistry and haematology. Several biomarkers were evaluated (*Supporting Information* *S4*), and no significant difference between patients receiving supplement and controls was identified for most of the biomarkers, except for blood urea nitrogen, albumin, and uric acid. Overall, HMB/Arg/Gln supplementation increased blood urea nitrogen,^45^, ^49^, ^53^ with changes differing between experimental groups in one study (*P* < 0.01)^45^ but not in another.^53^ Albumin increased,^45^, ^54^, ^58^ decreased,^53^ or was maintained,^56^ with one study reporting differences between groups.^53^ Uric acid increased in the experimental group, but decreased in the control group (*P* < 0.05).^45^ Furthermore, the supplementation was associated with a reduction in total cholesterol and triglycerides in one RCT^45^ but increases in total cholesterol in one NRSI.^58^

### Potential confounding effects

#### Adherence

Supplement adherence data were compiled in nine studies, including four RCTs^46^, ^47^, ^55^, ^56^ and five NRSIs^48^, ^49^, ^50^, ^51^, ^59^ (*Table* 1). Of these, three described the method used to assess adherence, which included self‐reported consumption of supplement through the use of logging and sheets.^49^, ^50^, ^56^ Adherence to the study protocol ranged from 83.0% to 93.8% in RCTs^46^, ^55^, ^56^ and from 91.4% to 98.2% in NRSIs.^48^, ^51^, ^59^ Poor palatability or refusal to consume the experimental supplement was described in two studies using HMB/Arg/Gln^46^, ^48^ and one study providing patients with a combination of HMB/Arg/Gln and HMB‐enriched ONS.^59^

#### Total energy and protein intakes

Two RCTs^44^, ^56^ and three NRSIs^49^, ^50^, ^59^ estimated both total energy and protein intakes (*Figures* S9 and S10). Methods of dietary assessment were diverse, including 3 day dietary record, multipass 24 h recall, food frequency questionnaire, food diaries completed during hospital stay, and daily self‐assessment sheets. Studies showed either increased^44^, ^50^ or decreased^49^, ^56^ energy intakes at follow‐up, but a consistently reduced daily protein intake (three of four studies). Furthermore, the minimum target caloric intake of 20 kcal/kg/day was reached at 4.1 ± 2.7 postoperative days (POD) in one NRSI^59^; protein intake at follow‐up (POD 3) was 0.81 ± 0.52 g/kg of body weight/day.^59^

#### Subgroup analysis

Subgroup analysis performed by Berk *et al*.^55^ showed a positive effect of the intervention only among patients with a 2–5% but not with a 5–10% weight loss; no differences were observed between cancer therapy, primary cancer site, metastases, sex, or ethnic groups. Furthermore, another study found that patients who adhered to an intervention with HMB alone for a period ≥3 weeks had greater increases in FFM but not on skeletal muscle index compared with those consuming the HMB supplement for a shorter period (*P* = 0.029 and *P* = 0.10, respectively).^50^

**Figure 3:**
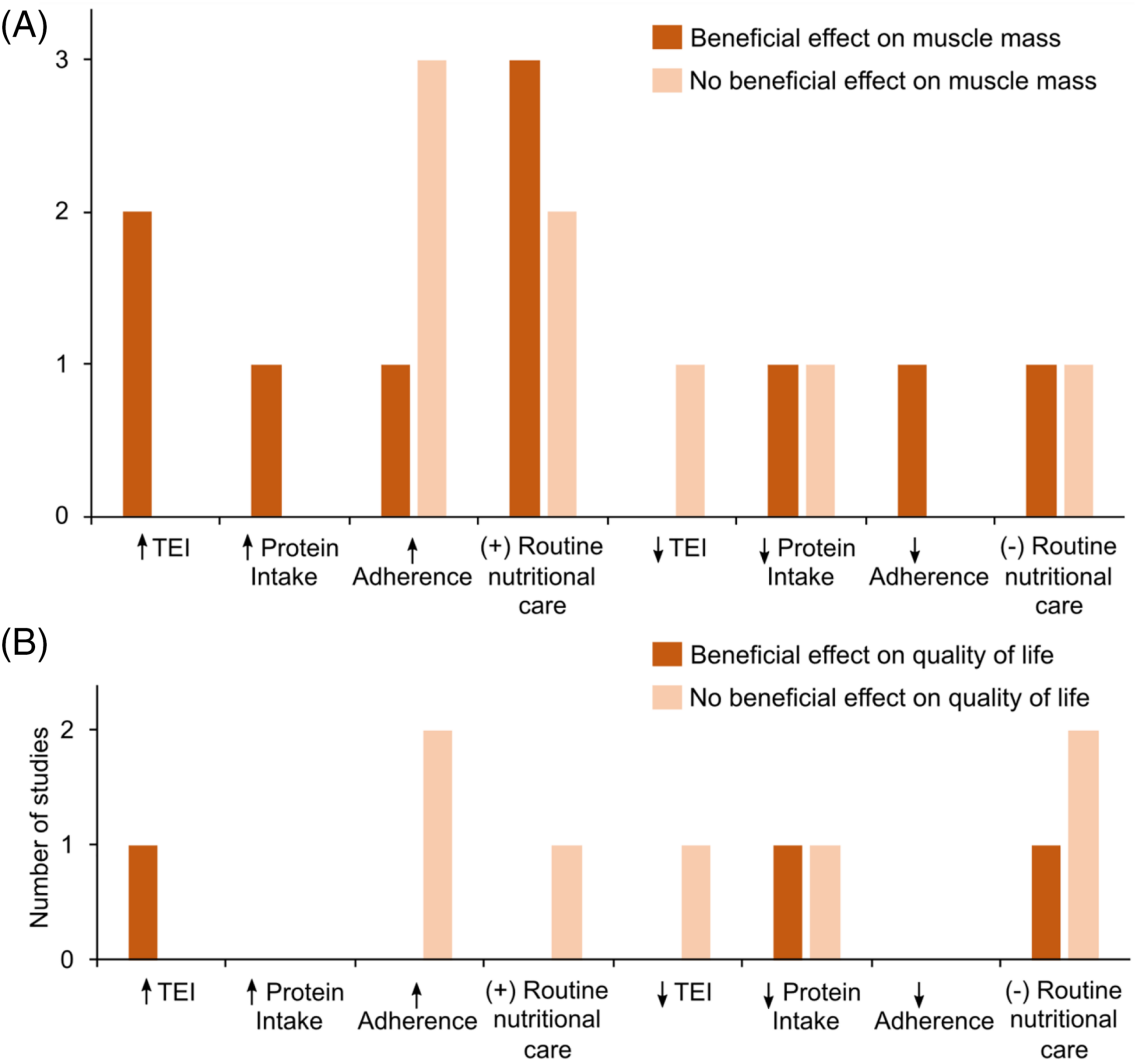
Bar graphs depicting the number of studies reporting the effects of β‐hydroxy β‐methylbutyrate (HMB) supplementation on (A) muscle mass and (B) quality of life according to changes in total energy (TEI) and protein intakes, adherence to the intervention protocol, and presence of nutritional co‐intervention (i.e. routine nutritional care). ↑, increased/high; ↓, decreased/low; (+), presence; (−), absence.

*Figures* 3 and S11 illustrate the effects of HMB supplementation on muscle mass and quality of life stratified by adherence to the intervention, concurrent nutritional care, and changes in total energy and protein intakes over the study course. Only a small number of studies had data available for analysis, and therefore, mixed findings were observed. One exception was related to total energy intake; HMB had a beneficial effect on muscle mass only in those studies in which participants had a greater total energy intake at follow‐up compared with baseline, and no beneficial effect in those studies with reductions in energy intake over the study course. Supplements containing HMB showed no beneficial effect on quality of life independent of adherence, routine nutritional care, and changes in energy and protein intakes.

### Risk of bias and sensitivity analysis

Risk of bias was low across all outcome domains in three studies and high in four studies (*Figures* S12 and S13). Most RCTs with a high risk of bias presented evidence of deviating from intended intervention or bias in outcome measurement. For example, studies assessing muscle mass with techniques known to have poor validity (e.g. foot‐to‐foot BIA and anthropometric measures) were rated as having a high risk of bias. Most NRSIs presented with high risk of bias due to the lack of control for potential baseline confounding.

**Figure 4:**
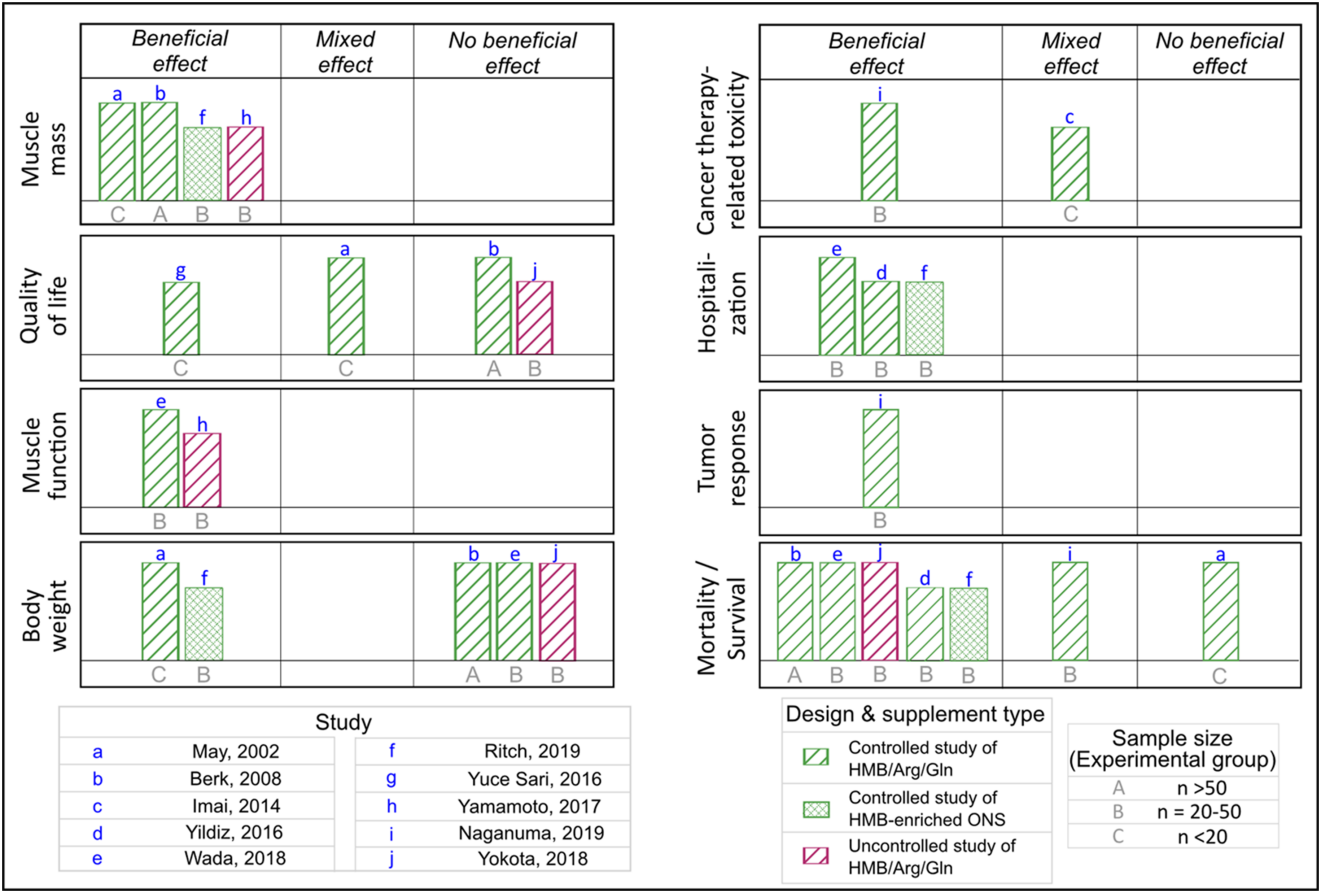
Summary of the evidence from higher‐quality studies on the effects of β‐hydroxy β‐methylbutyrate (HMB) supplementation on health outcomes of patients with cancer. Direction of effects was determined based on the study design. Differences in change between experimental and control groups were evaluated in controlled studies, and change from baseline to follow‐up within intervention group in uncontrolled studies. The height of the bar describes the study quality, with taller bars indicating low risk of bias, mid‐height bars representing moderate risk of bias, and shorter bars illustrating high risk of bias. Each lowercase letter represents a distinct study presented in the figure, and uppercase letters indicate sample size of experimental groups. Study design and supplement type are depicted by different colour and hatch patterns, respectively. As an example of interpretation, the controlled study by May *et al*. administered HMB/Arg/Gln to <20 patients in the experimental arm; compared with control group, the study found that supplements containing HMB had a beneficial effect on muscle mass and body weight, mixed effect on quality of life, and no beneficial effect on survival. Arg, arginine; Gln, glutamine; ONS, oral nutritional supplement.

*Figure* 4 depicts the effects of HMB supplements on muscle mass, quality of life, and other outcomes reported in higher‐quality studies. Of the outcomes assessed in ≥3 of these studies, evidence of improved muscle mass was found in four of four studies, decreased hospitalization‐related outcomes in three of three, and improved survival in five of seven. In contrast, no beneficial effects of HMB supplementation on quality of life and body weight were found in two of four and three of five studies, respectively. There were a limited number of higher‐quality studies evaluating the impact of HMB on muscle function, cancer therapy‐related toxicity, hospitalization, inflammation, and tumour response.

## Discussion

**Figure 5:**
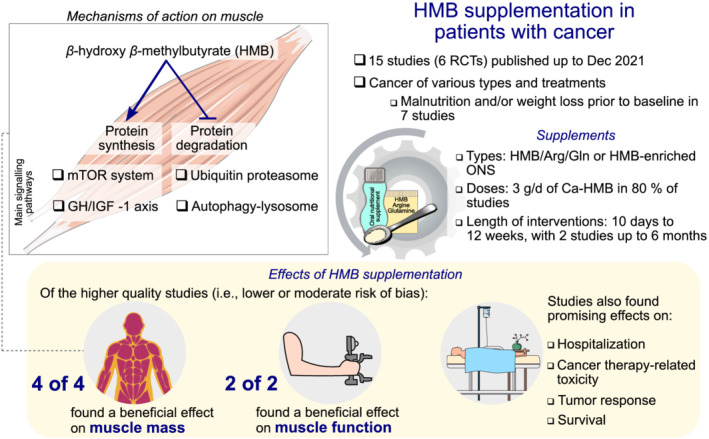
Graphical abstract illustrating the mechanisms of action of β‐hydroxy β‐methylbutyrate (HMB) on muscle and overall findings of this systematic review on the effects of HMB supplementation in patients with cancer.

This systematic review synthesizes the effects and safety of HMB supplementation on muscle mass and function, quality of life, and other clinical outcomes in patients with cancer (*Figure* 5). We found evidence that HMB supplementation increases muscle mass in four of four higher‐quality studies (i.e. low or moderate risk of bias), although the magnitude of changes was usually small. As metabolic alterations resulting from increased tumour energetic demands, a pro‐inflammatory state, and active therapy lead to progressive wasting and dramatic changes in body composition, HMB supplementation may mitigate additional muscle loss.^60^, ^61^ The effect of HMB on preserving muscle mass is probably tied to its known mechanism of action on down‐regulating the increase in muscle proteolysis driven by *NFκb* activation during cancer and other catabolic conditions,^27^, ^28^, ^62^ in addition to its role in stimulating muscle protein synthesis.^24^, ^25^, ^26^

The most studied dose of HMB in oncology is 3.0 g/day delivered as a Ca‐HMB (equivalent to 2.4 g/day of HMB); however, three of the studies evaluated a lower dose of 1.5 g/day of Ca‐HMB and reported some benefits on muscle function,^47^ tumour response,^48^ and survival.^47^, ^48^, ^59^ Based on this, the therapeutic dose of Ca‐HMB appears to range from 1.5 to 3.0 g/day. This is in line with what has been reported previously in other populations, with the most widely studied dose of 3.0 g/day,^23^ but evidence suggests a lower dose of 1.5 g/day of Ca‐HMB delivered as part of an ONS to have muscle health benefits.^63^, ^64^

### HMB and body weight

Most studies (three of five higher‐quality studies) reported no beneficial effects of HMB interventions on body weight. Although adequate energy and protein intakes are key factors for maintenance of muscle health and body weight,^65^, ^66^ only five studies assessed dietary intake throughout the study.^44^, ^49^, ^50^, ^56^, ^59^ In addition, a number of studies also included participants with malnutrition (or risk of malnutrition)^51^, ^53^, ^57^, ^58^, ^59^ or substantial weight loss prior to baseline assessment.^44^, ^52^, ^55^, ^57^, ^59^ It is possible that loss of body weight (and muscle mass) following HMB supplementation (as reported in some studies) may be explained by suboptimal nutritional status at baseline and/or inadequate overall dietary intake throughout the study, common confounding factors consistently described in other systematic reviews including in patients with cancer.^67^, ^68^, ^69^

Our subgroup analyses showed that potential factors contributing to the increase in muscle mass in the experimental arms could be in fact due to greater energy intake at follow‐up, as well as adherence to the intervention for a period longer than 3 weeks.^50^, ^55^ Notably, patients with substantial weight loss may also have cachexia or refractory cachexia and, therefore, limited anabolic potential.^15^, ^17^, ^70^ Further studies are needed to evaluate the effects of HMB where provision of caloric/macronutrient needs is optimized, while recruiting patients earlier on in the disease trajectory may maximize the anabolic potential also impacting nutritional status.^15^, ^17^, ^70^ Alternatively, supplementation with HMB‐enriched ONS may help patients meet their daily requirements as it provides additional source of calories and essential macronutrients and micronutrients.^71^ However, taste and smell alterations are common in these patients and may lead to poor adherence to the intervention protocol if specific strategies to encourage adherence are not incorporated throughout the study duration.^72^ Concurrent nutritional counselling is also needed to help patients achieve ideal energy and protein intakes.^30^, ^68^, ^73^, ^74^

### Relation between muscle and quality of life

Previous studies suggested a link between low muscle mass or function and quality of life in older adults as well as patients with cancer.^14^, ^75^ One potential explanation is that ‘healthier’ muscles may predict a better functional capacity, which is a core domain of quality of life. Although we found some evidence of the beneficial effects of HMB on muscle mass and strength (in higher‐quality studies evaluated), there was no benefit on quality of life. Notably, not all studies evaluated body composition and quality of life concurrently. As patients with cancer may experience physiological distress due to cancer treatment, tumour‐related symptoms, and other issues, quality of life could have been affected by factors beyond poor muscle health alone.^76^, ^77^ For example, fatigue was greater at the end of the intervention in both experimental and control groups in two studies.^52^, ^55^

### HMB and other outcome measures

Additional health outcomes of increasing value in nutritional oncology include reducing cancer therapy‐related toxicity and postoperative complications, as low muscle mass may decrease the tolerance to drug and surgical treatments.^78^, ^79^, ^80^ A 2021 meta‐analysis of 48 studies reported that patients with cancer with low muscle mass had two times greater odds of developing treatment toxicity.^6^ In spite of the limited number of studies, our findings suggest that supplementation with HMB/Arg/Gln had a beneficial impact on the incidence of some side effects such as dermatitis, oral mucositis, hand–foot skin reactions, and diarrhoea.^46^, ^48^, ^52^ Potential beneficial effects of HMB supplementation in surgical complications were only observed in studies providing both preoperative and postoperative HMB supplementation (rather than preoperatively alone), although the number of studies was small.^47^, ^54^, ^56^

Noteworthy, some of the non‐muscle‐related benefits observed in some studies may be attributed to the presence of arginine and glutamine in the supplement. Arginine and glutamine are considered immunonutrients associated with improved immune function.^81^ Because the immune system may be affected by cancer therapy, immunonutrients may have beneficial effects on toxicity, complications, and hospitalization. A previous meta‐analysis in patients with head and neck cancer reported that glutamine administration led to a reduced risk of developing Grade 2–4 mucositis during chemotherapy,^82^ and arginine supplementation reduced the incidence of complications and length of stay following surgery.^83^

### HMB safety in patients with cancer

Supplementation with HMB, independent of the form, appears to be safe and well tolerated in patients with cancer. None of the included studies reported serious adverse events directly related to this supplementation. Although some studies reported minor gastrointestinal events, it may be challenging to distinguish them from the effects of cancer therapy.^84^, ^85^ Furthermore, changes in blood chemistry and haematology were overall within an expected variation range. Although there was some evidence that supplementation with HMB/Arg/Gln may increase blood urea nitrogen in three studies,^45^, ^49^, ^53^ which could well be related to the increased Arg and Gln intake, only a small non‐significant increase in creatinine in both experimental and control groups was found in the only study measuring it.^45^

In muscle, the mTOR signalling pathway is the predominant metabolic pathway involved in muscle protein accretion.^86^ Both nutrients and exercise are transient stimulators of the mTOR pathway in muscle leading to muscle anabolism.^87^ HMB also transiently stimulates this pathway in muscle, leading to increased muscle protein synthesis.^24^ It is important to distinguish the normal metabolic stimulation of mTOR in muscle cells from what occurs in cancer cells. In certain cancer cells, the mTOR signalling pathway is chronically hyperactivated leading to tumour growth.^88^ However, this chronic hyperactivation of mTOR signalling is due to genetic mutations of either mTOR or related genes in the signalling cascade, leading to constitutive expression of these proteins that do not respond to the inhibitory processes in place that occurs under normal conditions.^88^

The benefits of exercise and protein, both transient activators of mTOR in muscle, are well evidenced in patients with cancer.^68^, ^89^, ^90^ In fact, exercise and adequate nutrient intake, especially high‐protein intake, are recommended to counter malnutrition and muscle loss that occurs over the course of the disease.^74^ Similarly, HMB studies summarized in this review demonstrate a benefit for muscle health (i.e. muscle mass and function), with a good safety profile. Of the included studies, one assessed impact on tumour growth rate with no significant differences found between the experimental group and historical controls.^48^ Notably, previous animal studies demonstrated a benefit of HMB in attenuating tumour growth rates.^27^, ^91^, ^92^

### The importance of optimizing nutritional status in future studies

Given the potential yet limited evidence on the effects of HMB supplementation in patients with cancer, future studies are needed to advance our understanding. A recent scoping review of registered clinical trials investigating nutrition interventions targeting muscle‐related outcomes revealed that 3 of 22 RCTs are evaluating the effects of HMB supplementation in this population.^93^ Of these ongoing studies, dietary advice with the goal of achieving a high‐protein diet is provided in one but none are investigating the effects of HMB‐enriched ONS. Notably, it is also unclear if other strategies are being used to optimize nutritional status in these studies, which may make it difficult to fully evaluate the effect of HMB in mitigating muscle and function loss, especially in patients with cancer who develop malnutrition along the course of the study. Adaptive clinical trials may be superior to traditional RCTs as they allow for protocol modifications to address nutritional needs of study patients.^94^, ^95^ Continuous evaluation of nutritional status, dietary intake, and adherence to the intervention is also essential to ensure high‐quality evidence, as recommended in previous systematic reviews of nutrition interventions in oncology.^67^, ^68^

### Strengths and limitations

Although we reported findings from all included studies, our conclusions were formulated solely based on the best available evidence. We used a robust approach to reduce publication bias by including only higher‐quality RCTs and NRSIs in the sensitivity analysis. A high heterogeneity of methods to assess and report outcome measures precluded a meta‐analysis. As such, we chose to perform a synthesis without meta‐analysis rather than reporting each study using a narrative style.^33^ The primary approach to synthesize data was vote counting. Thus, findings from our systematic review should be interpreted considering the limitations of this method, as it was solely based on the direction of effect. As such, there is no information of the combined magnitude of the effect.^38^ Although several systematic reviews still use vote counting based on the statistical significance, the Cochrane Handbook for Systematic Reviews of Intervention states that this is an ‘unacceptable synthesis method’ as it can lead to erroneous conclusions from underpowered studies.^38^ The recommendation is therefore to combine the vote‐counting method with a sign test.^38^ As the sign test could be problematic for a small group of studies due to lack of power, we opted not to use this test in our review,^96^ and thus reported the effect sizes within each outcome and *P*‐values in summary figures.

## Conclusions

This comprehensive systematic review found some evidence of a beneficial effect of HMB supplementation on muscle mass, function, hospitalization outcomes, and survival but not on quality of life and body weight in patients with cancer. As a limited number of high‐quality studies were included, our findings highlight the need for more well‐designed RCTs to further explore the benefits of HMB supplementation in patients with cancer. Suggestions for future studies include use of more sophisticated techniques for muscle mass assessment vs. anthropometric measures as a primary outcome, reporting whether changes in outcomes are above the minimally detectable change of the method (i.e. are not a result of measurement errors), recording adherence and dietary intake as well as considerations on approaches to improve patient adherence, reporting (and correcting) patient's nutritional status (i.e. presence of malnutrition), stratifying the analysis by cancer stage and type, and malnutrition status. Moreover, future clinical trials should always evaluate the effects of HMB supplementation (as with any other nutrition intervention) on patient‐reported outcomes.^97^

## Conflict of interest

This is an investigator‐initiated work by C.M.P. C.M.P. has previously received honoraria and/or paid consultancy from Abbott Nutrition, Nutricia, Nestlé Health Science, Fresenius Kabi, Pfizer, and Helsinn. C.E.O. has received honoraria from Abbott Nutrition for assisting C.M.P. with writing of the manuscript. S.L.P. is employed by Abbott Nutrition. N.E.D. has previously received grant funding and paid consultancy from Abbott Nutrition. P.J.A. has previously received grant funding and honoraria from Abbott Nutrition and Fresenius Kabi.

## Funding

C.M.P. is supported by a Canadian Institutes of Health Research (CIHR) New Investigator Salary Award and the Campus Alberta Innovation Research Chair Program.

## Supporting information


**Supporting Information S1.** Search Strategy
**Supporting Information S2.** Eligibility criteria of studies
**Supporting Information S3.** Frequency of adverse events reported in studies administering β‐hydroxy β‐methylbutyrate (HMB) supplements in patients with cancer
**Supporting Information S4.** Changes in blood chemistry and hematology within experimental (EG) and control groups (CG) in randomized controlled trials and non‐randomized studies of intervention with β‐hydroxy β‐methylbutyrate (HMB) in patients with cancer

**Figure S1.** Effect direction plot summarizing direction of health impacts from studies administering *β*‐hydroxy *β*‐methylbutyrate (HMB) supplements in patients with cancer. Effect direction: upward arrow ▲= beneficial health impact, downward arrow ▼= no beneficial health impact, sideways arrow ◄►= mixed effects. Sample size: Final sample size (individuals) in intervention group as large arrow ▲ >50; medium arrow ▲ 20‐50; small arrow ▲ <20. Study quality: denoted by row colour: green = low risk of bias; amber = some concerns; red = high risk of bias. Type of statistical test: A = change from baseline to follow‐up within the experimental (i.e., intervention) arm; B = difference in change between experimental and control groups; C = difference between experimental and control groups at follow‐up in controlled studies, and difference between baseline to follow‐up within the experimental group in uncontrolled studies. * May et al. 2002 and Rathmacher et al. 2004 described findings from the same study. Abbreviations: Arg, arginine; Gln, glutamine; HMB, *β*‐hydroxy *β*‐methylbutyrate; QofL, health‐related quality of life; ONS, oral nutritional supplement; RCT, Randomised Controlled Trial.
**Figure S2.** Changes in muscle mass parameters from baseline to follow‐up within experimental (green bar) and control (gray bar) groups. Δ* indicates absolute or percent mean change, and Δ† represents absolute or percent median change. *P*‐values reported in black correspond to testing of changes from baseline to follow‐up. *P*‐values reported in blue and positioned on the right side of figure represent testing of differences between experimental and control groups. Abbreviations: Arg, arginine; BIA, bioelectrical impedance analysis; CT, computed tomography; FFM, fat‐free mass; Gln, glutamine; HMB, *β*‐hydroxy *β*‐methylbutyrate; NR, not reported; NS, not significant; ONS, oral nutritional supplement; ↓RoB, low risk of bias; ↔ RoB, moderate risk of bias; ↑ RoB, high risk of bias.
**Figure S3.** Changes in quality of life scores from baseline to follow‐up within experimental (green bar) and control (gray bar) groups. Δ* indicates absolute or percent mean change, and Δ† represents absolute or percent median change. *P*‐values reported in black correspond to testing of changes from baseline to follow‐up. *P*‐values reported in blue represent testing of differences between experimental and control groups. Abbreviations: Arg, arginine; Gln, glutamine; HMB, *β*‐hydroxy *β*‐methylbutyrate; ONS, oral nutritional supplement; ↓RoB, low risk of bias; ↔ RoB, moderate risk of bias; ↑ RoB, high risk of bias.
**Figure S4.** Changes in measures of muscle function from baseline to follow‐up within experimental (green bar) and control (gray bar) groups. Δ* indicates absolute or percent mean change, and Δ† represents absolute or percent median change. *P*‐values reported in black correspond to testing of changes from baseline to follow‐up. *P*‐values reported in blue represent testing of differences between experimental and control groups. Abbreviations: Arg, arginine; Gln, glutamine; HMB, *β*‐hydroxy *β*‐methylbutyrate; ↓RoB, low risk of bias; ↔ RoB, moderate risk of bias; ↑ RoB, high risk of bias.
**Figure S5.** Changes in measures of body weight and body mass index (BMI) from baseline to follow‐up within experimental (green bar) and control (gray bar) groups. Δ* indicates absolute or percent mean change, and Δ† represents absolute or percent median change. *P*‐values reported in black correspond to testing of changes from baseline to follow‐up. *P*‐values reported in blue represent testing of differences between experimental and control groups. Abbreviations: Arg, arginine; Gln, glutamine; HMB, *β*‐hydroxy *β*‐methylbutyrate; NR, not reported; ONS, oral nutritional supplement; ↓RoB, low risk of bias; ↔ RoB, moderate risk of bias; ↑ RoB, high risk of bias.
**Figure S6.** Incidence (in percent) of cancer therapy‐related toxicities at follow‐up within experimental (green bar) and control (gray bar) groups. *P*‐values represent testing of differences between experimental and control groups. Abbreviations: Arg, arginine; Gln, glutamine; HMB, *β*‐hydroxy *β*‐methylbutyrate; ONS, oral nutritional supplement; ↓RoB, low risk of bias; ↔ RoB, moderate risk of bias; ↑ RoB, high risk of bias.
**Figure S7.** Hospital length of stay and rate of readmissions and complications at follow‐up within experimental (green bar) and control (gray bar) groups. *P*‐values represent testing of differences between experimental and control groups. Abbreviations: Arg, arginine; Gln, glutamine; HMB, *β*‐hydroxy *β*‐methylbutyrate; ONS, oral nutritional supplement; ↓RoB, low risk of bias; ↔ RoB, moderate risk of bias; ↑ RoB, high risk of bias.
**Figure S8.** Mortality and survival rates at follow‐up within experimental (green bar) and control (gray bar) groups. *P*‐values represent testing of differences between experimental and control groups. Studies with data not shown reported zero deaths during the period described in the graph. Abbreviations: Arg, arginine; Gln, glutamine; HMB, *β*‐hydroxy *β*‐methylbutyrate; ONS, oral nutritional supplement; ↓RoB, low risk of bias; ↔ RoB, moderate risk of bias; ↑ RoB, high risk of bias.
**Figure S9.** Changes in energy intake from baseline to follow‐up within experimental (green bar) and control (gray bar) groups. *P*‐values represent testing of differences between experimental and control groups. Δ* indicates absolute or percent mean change, and Δ† represents absolute median change. Abbreviations: Arg, arginine; Gln, glutamine; HMB, *β*‐hydroxy *β*‐methylbutyrate; ONS, oral nutritional supplement; ↓RoB, low risk of bias; ↔ RoB, moderate risk of bias; ↑ RoB, high risk of bias.
**Figure S10.** Changes in protein intake from baseline to follow‐up within experimental (green bar) and control (gray bar) groups. *P*‐values represent testing of differences between experimental and control groups. Δ* indicates absolute or percent mean change, and Δ† represents absolute median change. Abbreviations: Arg, arginine; Gln, glutamine; HMB, *β*‐hydroxy *β*‐methylbutyrate; ONS, oral nutritional supplement; ↓RoB, low risk of bias; ↔ RoB, moderate risk of bias; ↑ RoB, high risk of bias.
**Figure S11.** Colormap depicting the number of studies reporting the effects of β‐hydroxy β‐methylbutyrate (HMB) supplementation on muscle mass and quality of life according to changes in total energy and protein intakes, adherence to the intervention protocol, and presence of nutrition co‐intervention (i.e., routine nutritional care). Effect direction: upward arrow ▲= beneficial effect, downward arrow ▼= no beneficial effect.
**Figure S12.** Risk of bias assessment of randomized controlled trials, stratified by study, outcomes, and domains. D1: Bias arising from the randomization process; D2: Bias due to deviations from intended intervention; D3: Bias due to missing outcome data; D4: Bias in measurement of the outcome; D5: Bias in selection of the reported result.
**Figure S13.** Risk of bias assessment of non‐randomized studies of intervention, stratified by study, outcomes, and domains. D1: Bias due to confounding; D2: Bias due to selection of participants; D3: Bias in classification of interventions; D4: Bias due to deviations from intended interventions; D5: Bias due to missing data; D6: Bias in measurement of outcomes; D7: Bias in selection of the reported result.
