# Supplementary material for: Effects of β‐hydroxy β‐methylbutyrate (HMB) supplementation on muscle mass, function, and other outcomes in patients with cancer: a systematic review
Source: J Cachexia Sarcopenia Muscle. 2022 Mar 17;13(3):1623–41. doi: 10.1002/jcsm.12952 (PMC9178154; doi:10.1002/jcsm.12952)
Supplement: Supplementary file 1 — Supporting Information S1. Search Strategy Supporting Information S2. Eligibility criteria of studies Supporting Information S3. Frequency of adverse events reported in studies administering β‐hydroxy β‐methylbutyrate (HMB) supplements in patients with cancer Supporting Information S4. Changes in blood chemistry and hematology within experimental (EG) and control groups (CG) in randomized controlled trials and non‐randomized studies of intervention with β‐hydroxy β‐methylbutyrate (HMB) in patients with cancer [file JCSM-13-1623-s001.pdf]

Supporting Information

Effects of *β*-hydroxy *β*-methylbutyrate (HMB) supplementation on muscle mass, function, and other outcomes in patients with cancer: a systematic review

Carla M. Prado[1*], Camila E. Orsso[1], Suzette L Pereira[2], Philip J. Atherton[3], Nicolaas E. P. Deutz[4]

[1] Human Nutrition Research Unit, Department of Agricultural, Food and Nutritional Science, University of Alberta, Canada

[2] Research and Development, Abbott Nutrition, United States

[3] Centre of Metabolism, Ageing & Physiology (COMAP), Medical Research Council (MRC) Versus Arthritis Centre for Musculoskeletal Ageing Research (CMAR) and National Institution for Health Research (NIHR) Biomedical Research Centre (BRC), University of Nottingham, United Kingdom

[4] Center for Translational Research in Aging & Longevity, Department of Health & Kinesiology, Texas A&M University, United States

***Corresponding author**
Professor Carla M. Prado, PhD, RD
2-021 Li Ka Shing Centre for Health Innovation
University of Alberta
Edmonton, AB, Canada T6G 2E1
Tel: 780.492.7934 / Fax: 780.492.9555
Email: Carla.prado@ualberta.ca

Supporting Information

Contents

Supporting Information S1 – Search Strategy 3

Supporting Information S2 – Eligibility criteria of studies. 7

Supporting Information S3 – Frequency of adverse events reported in studies administering β-hydroxy β-methylbutyrate (HMB) supplements in patients with cancer. 9

Supporting Information S4 – Changes in blood chemistry and hematology within experimental (EG) and control groups (CG) in randomized controlled trials and non-randomized studies of intervention with β-hydroxy β-methylbutyrate (HMB) in patients with cancer. 11

Supporting Information

Supporting Information S1 – Search Strategy

1. MEDLINE (via OVID)

Search date: First searched on Aug 16, 2021 and updated on Dec 12, 2021

Search results: 886 on Aug 16, 2021; 881 on Dec 12, 2021

No.	Search term
1	(HMB or beta-hydroxy-beta-methylbutyrate or hydroxymethylbutyrate).mp.
2	oral nutrition* supplement*.mp.
3	((protein* or energy) adj3 ONS).mp.
4	sip feed*.mp.
5	or/1-4
6	exp Neoplasms/
7	exp Carcinoma/
8	(cancer* or carcinoma* or tumo?r* or malignan* or metasta* or neoplasm* or oncolog*).mp.
9	(leukemia* or lymphoma* or melanoma* or sarcoma*).mp.
10	or/6-9
11	5 and 10
12	animals/
13	humans/
14	12 not (12 and 13)
15	(veterinary or rabbit or rabbits or animal or animals or mouse or mice or rodent or rodents or rat or rats or hamster* or pig or pigs or porcine or horse* or equine or cow or cows or bovine or goat or goats or sheep or ovine or canine or dog or dogs or feline or cat or cats or zebrafish).ti.
16	14 or 15
17	11 not 16
18	limit 17 to english language
19	limit 18 to case reports
20	18 not 19

2. CINAHL

Search date: First searched on Aug 16, 2021 and updated on Dec 12, 2021 (Expanders - Apply equivalent subjects; Search modes - Boolean/Phrase)

Search results: 165 on Aug 16, 2021; 153 on Dec 12, 2021

No.	Search term
S1	HMB OR "beta-hydroxy-beta-methylbutyrate" OR hydroxymethylbutyrate
S2	"oral nutrition* supplement*"
S3	(protein* OR energy) N3 ONS
S4	"sip feed*"

No.	Search term
**S5**	S1 OR S2 OR S3 OR S4
**S6**	(MH "Neoplasms+")
**S7**	(MH "Carcinoma+")
**S8**	cancer* OR carcinoma* OR tumo?r* OR malignan* OR metasta* OR neoplasm* OR oncolog*
**S9**	leukemia* OR lymphoma* OR melanoma* OR sarcoma*
**S10**	S6 OR S7 OR S8 OR S9
**S11**	S5 AND S10
**S12**	S5 AND S10 [Limit Publication Type: Case Study]
**S13**	S11 NOT S12
**S14**	S13 [Limit English language]
**S15**	TI veterinary OR rabbit OR rabbits OR animal OR animals OR mouse OR mice OR rodent OR rodents OR rat OR rats OR hamster* OR pig OR pigs OR porcine OR horse* OR equine OR cow OR cows OR bovine OR goat OR goats OR sheep OR ovine OR canine OR dog OR dogs OR feline OR cat OR cats OR zebrafish
**S16**	S14 NOT S15

3. Embase (via Elsevier)

Search date: First searched on Aug 16, 2021 and updated on Dec 12, 2021

Search results: 1,889 on Aug 16, 2021; 1950 on Dec 12, 2021

No.	Search term
**#1**	hmb OR 'beta-hydroxy-beta-methylbutyrate' OR hydroxymethylbutyrate
**#2**	'oral nutrition* supplement*'
**#3**	(protein* OR energy) NEAR/3 ('oral nutrition* supplement*' OR ons)
**#4**	'sip feed*'
**#5**	#1 OR #2 OR #3 OR #4
**#6**	'neoplasms'/exp
**#7**	'carcinoma'/exp
**#8**	cancer* OR carcinoma* OR tumo?r* OR malignan* OR metasta* OR neoplasm* OR oncolog*
**#9**	leukemia* OR lymphoma* OR melanoma* OR sarcoma*
**#10**	#6 OR #7 OR #8 OR #9
**#11**	#5 AND #10
**#12**	#11 NOT ('case report'/exp OR 'case study'/exp)
**#13**	veterinary OR rabbit OR rabbits OR animal OR animals OR mouse OR mice OR rodent OR rodents OR rat OR rats OR hamster* OR pig OR pigs OR porcine OR horse* OR equine OR cow OR cows OR bovine OR goat OR goats OR sheep OR ovine OR canine OR dog OR dogs OR feline OR cat OR cats OR zebrafish
**#14**	#12 NOT #13
**#15**	#14 AND [english]/lim

4. Cochrane CENTRAL

Search date: First searched on Aug 16, 2021 and updated on Dec 12, 2021

Search results: 255 on Aug 16, 2021; 270 on Dec 12, 2021

No.	Search term
#1	(HMB OR "beta-hydroxy-beta-methylbutyrate" OR hydroxymethylbutyrate OR "oral nutrition* supplement*" OR "sip feed*"):ti,ab,kw OR ((protein* OR energy) NEAR/3 ONS):ti,ab,kw (Word variations have been searched)
#2	(cancer* OR carcinoma* OR tumo?r* OR malignan* OR metasta* OR neoplasm* OR oncolog*):ti,ab,kw OR (leukemia* OR lymphoma* OR melanoma* OR sarcoma*):ti,ab,kw (Word variations have been searched)
#3	MeSH descriptor: [Neoplasms] explode all trees
#4	MeSH descriptor: [Carcinoma] explode all trees
#5	{OR #2-#4}
#6	#1 AND #5 [Limit Content Type: in Trials]

5. SCOPUS

Search date: First searched on Aug 16, 2021 and updated on Dec 12, 2021

Search results: 1,233 on Aug 16, 2021; 1,264 on Dec 12, 2021

No.	Search term
#1	(TITLE-ABS-KEY ((hmb OR "beta-hydroxy-beta-methylbutyrate" OR hydroxymethylbutyrate OR "oral nutrition* supplement*" OR "sip feed*")) AND TITLE-ABS-KEY (cancer* OR carcinoma* OR tumor* OR tumour* OR malignan* OR metasta* OR neoplasm* OR oncolog* OR leukemia* OR lymphoma* OR melanoma* OR sarcoma*) AND NOT TITLE-ABS-KEY (veterinary OR rabbit OR rabbits OR animal OR animals OR mouse OR mice OR rodent OR rodents OR rat OR rats OR hamster* OR pig OR pigs OR porcine OR horse* OR equine OR cow OR cows OR bovine OR goat OR goats OR sheep OR ovine OR canine OR dog OR dogs OR feline OR cat OR cats OR zebrafish OR {case study} OR {case report} OR {case series})) AND (LIMIT-TO (LANGUAGE , "English"))

6. Grey literature

Search date: First searched on Aug 16, 2021 and updated on Dec 12, 2021

Search results: 148 on Aug 16, 2021; 153 on Dec 12, 2021

Source Searched	Search terms
**Proquest Dissertations & Theses Global**	noft(hmb OR "beta-hydroxy-beta-methylbutyrate" OR hydroxymethylbutyrate OR "oral nutrition* supplement*" OR "sip feed*") AND noft(cancer OR cancer* OR carcinoma* OR tumor* OR tumour* OR malignan* OR metasta* OR neoplasm* OR oncolog* OR leukemia* OR lymphoma* OR melanoma* OR sarcoma* neoplasm) NOT noft(veterinary OR rabbit OR rabbits OR animal OR animals OR mouse OR mice OR rodent OR rodents OR rat OR rats OR hamster* OR pig OR pigs OR porcine OR horse* OR equine OR cow OR cows OR bovine OR goat OR goats OR sheep OR ovine OR canine OR dog OR dogs OR feline OR cat OR cats OR zebrafish OR "case study" OR "case report" OR "case series")
**Clinicaltrials.gov**	Condition or disease: neoplasm OR "malignant neoplasm" OR cancer OR carcinoma OR oncology<br>Other terms: HMB OR “beta-hydroxy-beta-methylbutyrate” OR hydroxymethylbutyrate OR "oral nutritional supplement"<br>Filters: Completed, interventional
**Google**	"cancer" AND beta-hydroxy-beta-methylbutyrate OR oral-nutritional-supplement AND -pubmed -wiley -sciencedirect -review

Supporting Information

Supporting Information S2 – Eligibility criteria of studies.

	**Inclusion criteria**	**Exclusion criteria**
**Participants / population**	- Adults (≥18 years) with active cancer of any type and stage<br>- Patients may be undergoing any type and phase of cancer treatment<br>- Inpatient (i.e., hospitalized) or outpatient settings<br>- Of any nutritional status	- Disease-free survivors of cancer<br>- Children and adolescents (<18 years)<br>- Pregnancy and lactation<br>- Healthy adults<br>- Animals
**Intervention**	- Dietary supplementation with HMB alone, combined with amino acids or other nutrients, or in ONS<br>- HMB of any dose and provided in any form<br>- Any forms of enteral feeding (i.e., oral and/or tube feeding)<br>- Interventions of any duration<br>- Patients may receive dietary counseling and/or participate in exercise interventions concurrently	- Food supplements without HMB<br>- ONS without HMB
**Comparator**	<u>Inclusion criteria applicable to **RCTs** only:</u><br>- Placebo (i.e., food supplements or oral nutritional supplements not containing HMB)<br>- Standard care (i.e., no nutritional intervention or dietary counseling alone without provision of food supplements or oral nutritional supplements containing HMB)<br><u>Inclusion criteria applicable to **NRCTs** only:</u><br>- Placebo (i.e., food supplements or oral nutritional supplements not containing HMB)<br>- Standard care (i.e., no nutritional intervention or dietary counseling alone without provision of food supplements or oral nutritional supplements containing HMB)<br>- Historical controls<br>**- No control group (i.e., single-arm study)**	Not applicable
**Outcomes**	- Muscle mass (assessed by any body composition technique or estimated by anthropometrics); quality of life (using validated questionnaires); body weight or BMI; muscle strength; physical performance; prevalence of low muscle mass or function, sarcopenia, or cachexia; inflammation markers; symptom assessment; changes in	Not applicable

	Inclusion criteria	Exclusion criteria
	organ and tissue function; cancer therapy related toxicities; survival; hospitalization rate, length of hospital stay, incidence of postoperative complications; markers of tumor growth or tumor response; adverse events; adherence	
**Study design**	- RCT<br>- NRSI (i.e., quasi-randomized controlled trials; clinical trials of single study arm; and observational studies, such as cohort and case-control studies)<br>- Of any sample size	- Reports not including original data (e.g., meta-analyses, systematic reviews, or study protocols)<br>- Theses and dissertations<br>- Studies terminated earlier than planned<br>- Abstracts from conference proceedings<br>- Case reports of any sample size
**Others**	- Full text article<br>- English Language	- Language other than English<br>- Abstract only

Abbreviations: BMI, body mass index; HMB, β-hydroxy β-methylbutyrate; NRSI, non-randomized study of intervention; ONS, oral nutritional supplements; RCT, randomized clinical trial.

Supporting Information S3 – Frequency of adverse events reported in studies administering β-hydroxy β-methylbutyrate (HMB) supplements in patients with cancer.

Reference	Supplement type	Target Ca-HMB dose (g/d)	Length of intervention	Adverse events
May, 2002 & Rathmacher, 2004	HMB/Arg/Gln	3.0	24 weeks	Dropout rate was not due to adverse events
Berk, 2008	HMB/Arg/Gln	3.0	8 weeks	17 patients (7%) had side effects (e.g., nausea, constipation, and/or diarrhea) possibly due to the nutrition intervention
Imai, 2014	HMB/Arg/Gln	3.0	From the first day of CCRT up to until 1 week after the completion of CCRT	3 patients (15%) had anorexia and nausea after the first course of chemotherapy
Naganuma, 2019	HMB/Arg/Gln	1.5	12 weeks	No adverse events related to supplement intake
Yamamoto, 2017	HMB/Arg/Gln[†]	3.0	16 days (range 7–26 days) preoperatively	No adverse events
Yokota, 2018	HMB/Arg/Gln	3.0	First day until the last day of radiotherapy	14.3% patients had grade ≥ 2 diarrhea
Saka, 2019	HMB/Arg/Gln	3.0	36 days	No serious adverse events related to supplement intake were reported
de Luis, 2018	HMB-enriched ONS	3.0	12 weeks	No serious adverse events were reported
Previtali, 2020	HMB-enriched ONS alone or withMB/Arg/Gln	1.5-6.0	Arm 1: 5 days preoperatively; Arm 2 & 3: 15 days preoperatively	1 patient (2.9%) had vomiting. No serious adverse events were reported

Reference	Supplement type	Target Ca-HMB dose (g/d)	Length of intervention	Adverse events
Parlak, 2020	HMB/Arg/Gln	3.0	Mean ± SD: 10.42 ± 5.73 days	1 patient (1.2%) had diarrhea; 5 patients (5.8%) had nausea and vomiting; 2 patients (2.3%) had abdominal distension

Abbreviations: Arg, arginine; CCRT, concurrent chemoradiotherapy; Gln, glutamine; HMB, β-hydroxy β-methylbutyrate; ONS, oral nutritional supplements; SD, standard deviation.

Supporting Information S4 – Changes in blood chemistry and hematology within experimental (EG) and control groups (CG) in randomized controlled trials and non-randomized studies of intervention with β-hydroxy β-methylbutyrate (HMB) in patients with cancer**.**

**First author, year**		**Rathmacher, 2004**	**Ritch, 2019**	**Parlak, 2020**	**Yokota, 2018**	**Saka, 2019**	**Wada, 2018**	**Yildiz, 2016**	**Cornejo-Pareja, 2021**
**Supplement type**		HMB/Arg/Gln	HMB-enriched ONS	HMB/Arg/Gln	HMB/Arg/Gln	HMB/Arg/Gln	HMB/Arg/Gln	HMB/Arg/Gln	HMB-enriched ONS
**Target HMB dose (g/d)**		3.0	3.0	3.0	3.0	3.0	1.5	3.0	3.0
**Δ-Blood urea nitrogen (mg/dL)**	EG	9.7		7.66	16.6				
	CG	-1.3		CG1: 6.36/ CG2: 6.19	NA				
	P-value	p <0.01		p ≥0.05	NA				
**Δ-Albumin (g/dL)**	EG	0.06	0	-0.06				0.10	0.8
	CG	-0.11	0	CG1: 0.26 / CG2: -0.16				0.04	
	P-value	p ≥0.05	0.92	0.004					p <0.05
**Δ-Prealbumin (g/dL)**	EG		-1.9			4.23		3.0	5.7
	CG		2.4			NA		1.9	
	P-value		0.73			NA			p <0.05
**Δ-creatine (mg/dL)**	EG			0.06					
	CG			CG1: -0.03 / CG2: 0.07					
	P-value			p ≥0.05					
	EG	0.16							
	CG	0.05							

**First author, year**		**Rathmacher, 2004**	**Ritch, 2019**	**Parlak, 2020**	**Yokota, 2018**	**Saka, 2019**	**Wada, 2018**	**Yildiz, 2016**	**Cornejo-Pareja, 2021**
**Δ-creatinine (mg/dL)**	*P*-value	p ≥0.05							
**Δ-Total cholesterol (mg/dL)**	EG	-24							24.6
	CG	12.4							
	P-value	p ≥0.05							p <0.05
**Δ-Triglycerides (mg/dL)**	EG	-61							
	CG	17.2							
	P-value	p ≥0.05							
**Δ-AST (U/L)**	EG			4.72					
	CG			CG1: 6.61 / CG2: 13.21					
	P-value			p ≥0.05					
**Δ-Glucose (mg/dL)**	EG	-10.5		4.04					
	CG	2.9		CG1: 4.78; CG2: 3.97					
	P-value	p ≥0.05		p ≥0.05					
**Δ-Growth hormone (ng/mL)**	EG						0.227 (-9.402, 4.902)		
	CG						-0.002 (-4.271, 1.444)		
	P-value						0.057		
	EG		6.4					10.0	
	CG		-4.2					3.0	

Supporting Information

First author, year		Rathmacher, 2004	Ritch, 2019	Parlak, 2020	Yokota, 2018	Saka, 2019	Wada, 2018	Yildiz, 2016	Cornejo-Pareja, 2021
**Δ-transferrin (ng/mL)**	*P*-value		0.95						
**Δ-lymphocyte**	EG	0.12		259.96					0.4
	CG	-0.37		CG1: 333.92/ CG2: -142.87					
	P-value	p ≥0.05		p ≥0.05					p <0.05
**Δ-white blood cell**	EG	-2.35							
	CG	-1.14							
	P-value	p ≥0.05							
**Δ-uric acid (mg/dL)**	EG	0.49							
	CG	-1.28							
	P-value	<0.05							

Abbreviations: Arg, arginine; CG, control group; EG, experimental group; Gln, glutamine; HMB, β-hydroxy β-methylbutyrate; ONS, oral nutritional supplements.
