# Supplementary material for: Effects of β‐hydroxy β‐methylbutyrate (HMB) supplementation on muscle mass, function, and other outcomes in patients with cancer: a systematic review
Source: J Cachexia Sarcopenia Muscle. 2022 Mar 17;13(3):1623–41. doi: 10.1002/jcsm.12952 (PMC9178154; doi:10.1002/jcsm.12952)
Supplement: Supplementary file 2 — Figure S1. Effect direction plot summarizing direction of health impacts from studies administering β‐hydroxy β‐methylbutyrate (HMB) supplements in patients with cancer. Effect direction: upward arrow ▲= beneficial health impact, downward arrow ▼= no beneficial health impact, sideways arrow ◄►= mixed effects. Sample size: Final sample size (individuals) in intervention group as large arrow ▲ >50; medium arrow ▲ 20‐50; small arrow ▲ <20. Study quality: denoted by row colour: green = low risk of bias; amber = some concerns; red = high risk of bias. Type of statistical test: A = change from baseline to follow‐up within the experimental (i.e., intervention) arm; B = difference in change between experimental and control groups; C = difference between experimental and control groups at follow‐up in controlled studies, and difference between baseline to follow‐up within the experimental group in uncontrolled studies. * May et al. 2002 and Rathmacher et al. 2004 described findings from the same study. Abbreviations: Arg, arginine; Gln, glutamine; HMB, β‐hydroxy β‐methylbutyrate; QofL, health‐related quality of life; ONS, oral nutritional supplement; RCT, Randomised Controlled Trial. Figure S2. Changes in muscle mass parameters from baseline to follow‐up within experimental (green bar) and control (gray bar) groups. Δ* indicates absolute or percent mean change, and Δ† represents absolute or percent median change. P‐values reported in black correspond to testing of changes from baseline to follow‐up. P‐values reported in blue and positioned on the right side of figure represent testing of differences between experimental and control groups. Abbreviations: Arg, arginine; BIA, bioelectrical impedance analysis; CT, computed tomography; FFM, fat‐free mass; Gln, glutamine; HMB, β‐hydroxy β‐methylbutyrate; NR, not reported; NS, not significant; ONS, oral nutritional supplement; ↓RoB, low risk of bias; ↔ RoB, moderate risk of bias; ↑ RoB, high risk of bias. Figure S3. Changes in qua [file JCSM-13-1623-s002.pptx]

## Slide 1
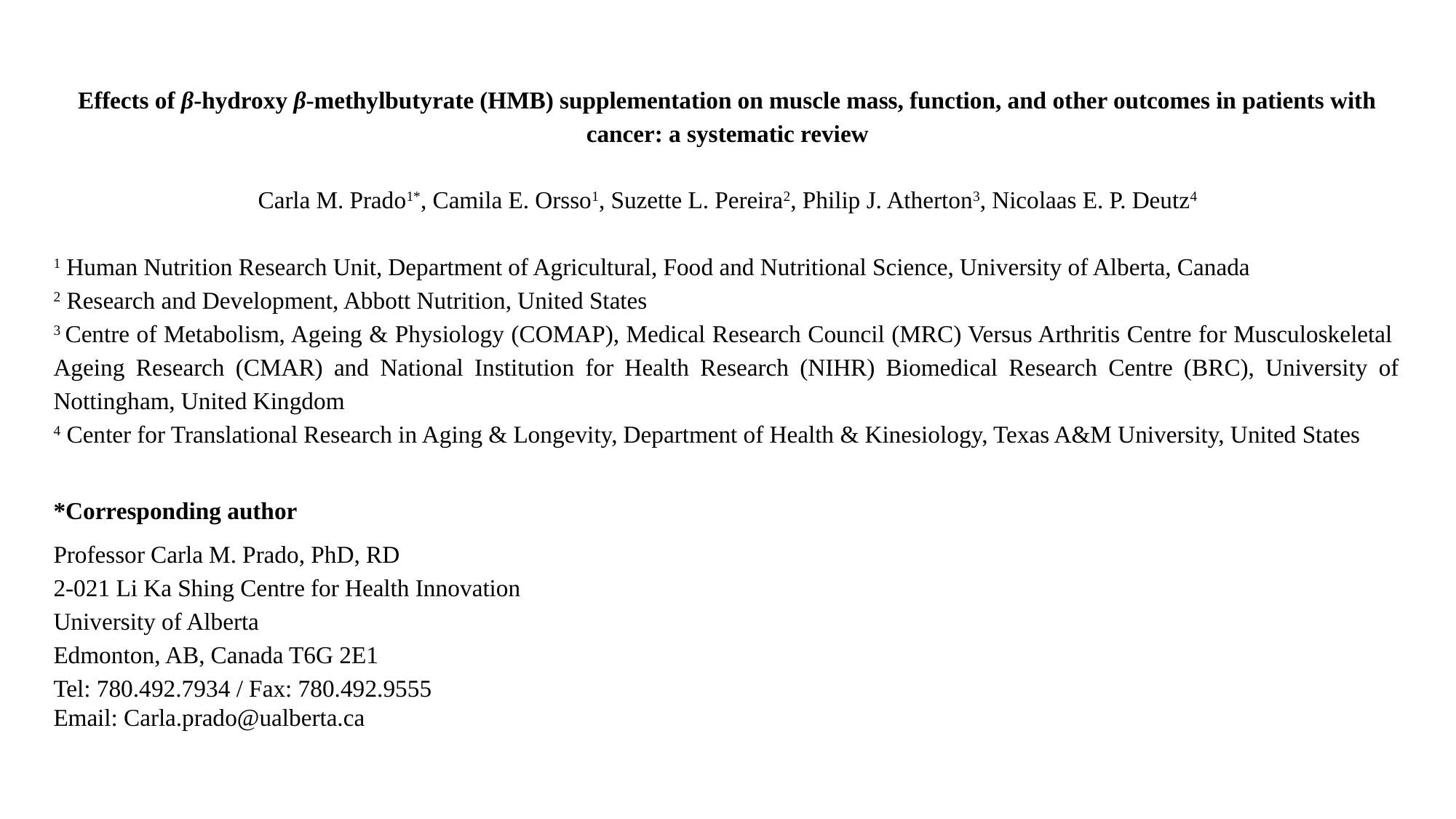

Effects of β-hydroxy β-methylbutyrate (HMB) supplementation on muscle mass, function, and other outcomes in patients with cancer: a systematic review
Carla M. Prado1*, Camila E. Orsso1, Suzette L. Pereira2, Philip J. Atherton3, Nicolaas E. P. Deutz4
1 Human Nutrition Research Unit, Department of Agricultural, Food and Nutritional Science, University of Alberta, Canada
2 Research and Development, Abbott Nutrition, United States
3 Centre of Metabolism, Ageing & Physiology (COMAP), Medical Research Council (MRC) Versus Arthritis Centre for Musculoskeletal Ageing Research (CMAR) and National Institution for Health Research (NIHR) Biomedical Research Centre (BRC), University of Nottingham, United Kingdom
4 Center for Translational Research in Aging & Longevity, Department of Health & Kinesiology, Texas A&M University, United States
*Corresponding author
Professor Carla M. Prado, PhD, RD
2-021 Li Ka Shing Centre for Health Innovation
University of Alberta
Edmonton, AB, Canada T6G 2E1
Tel: 780.492.7934 / Fax: 780.492.9555
Email: Carla.prado@ualberta.ca

## Slide 2
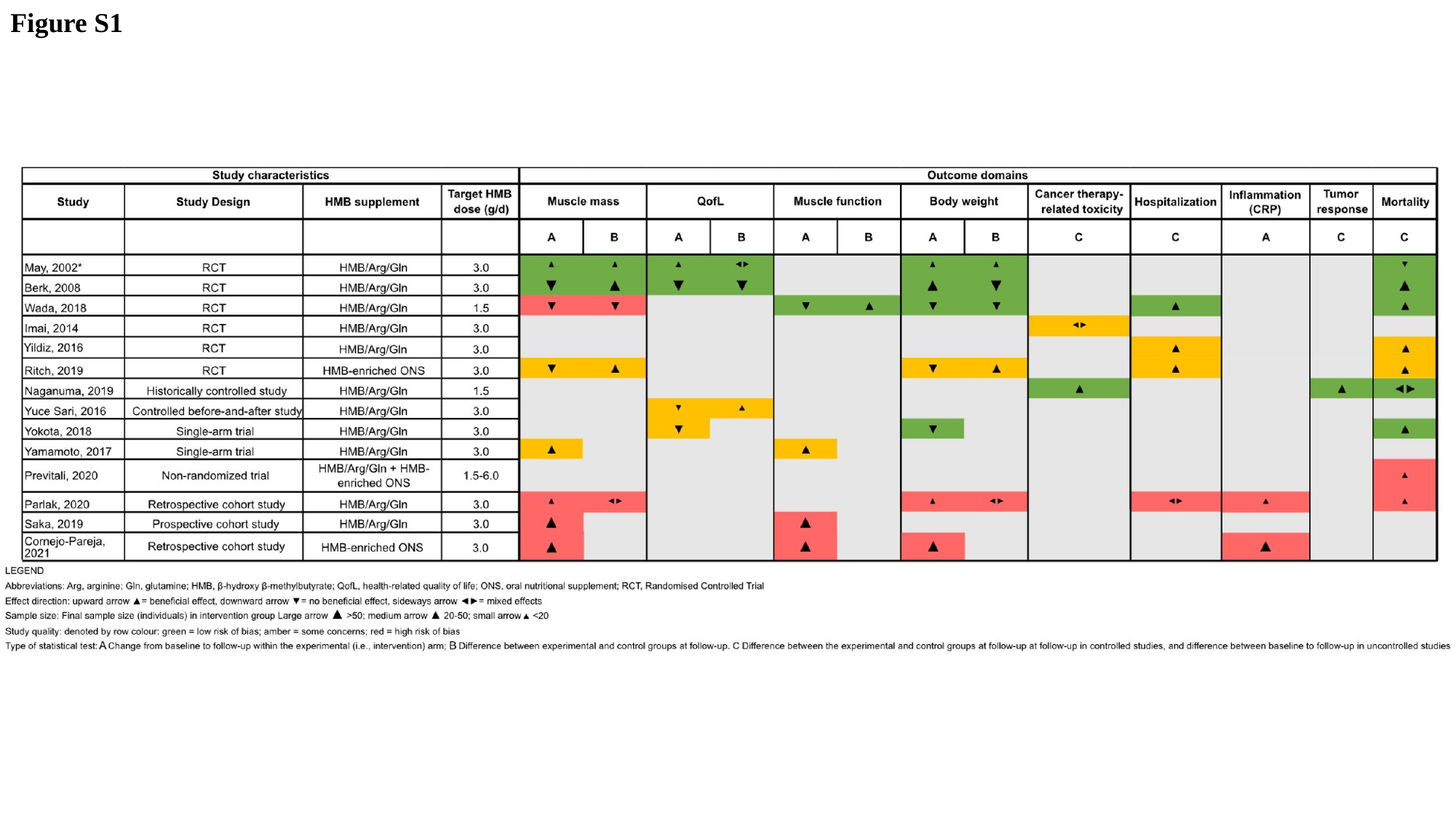

Figure S1

## Slide 3
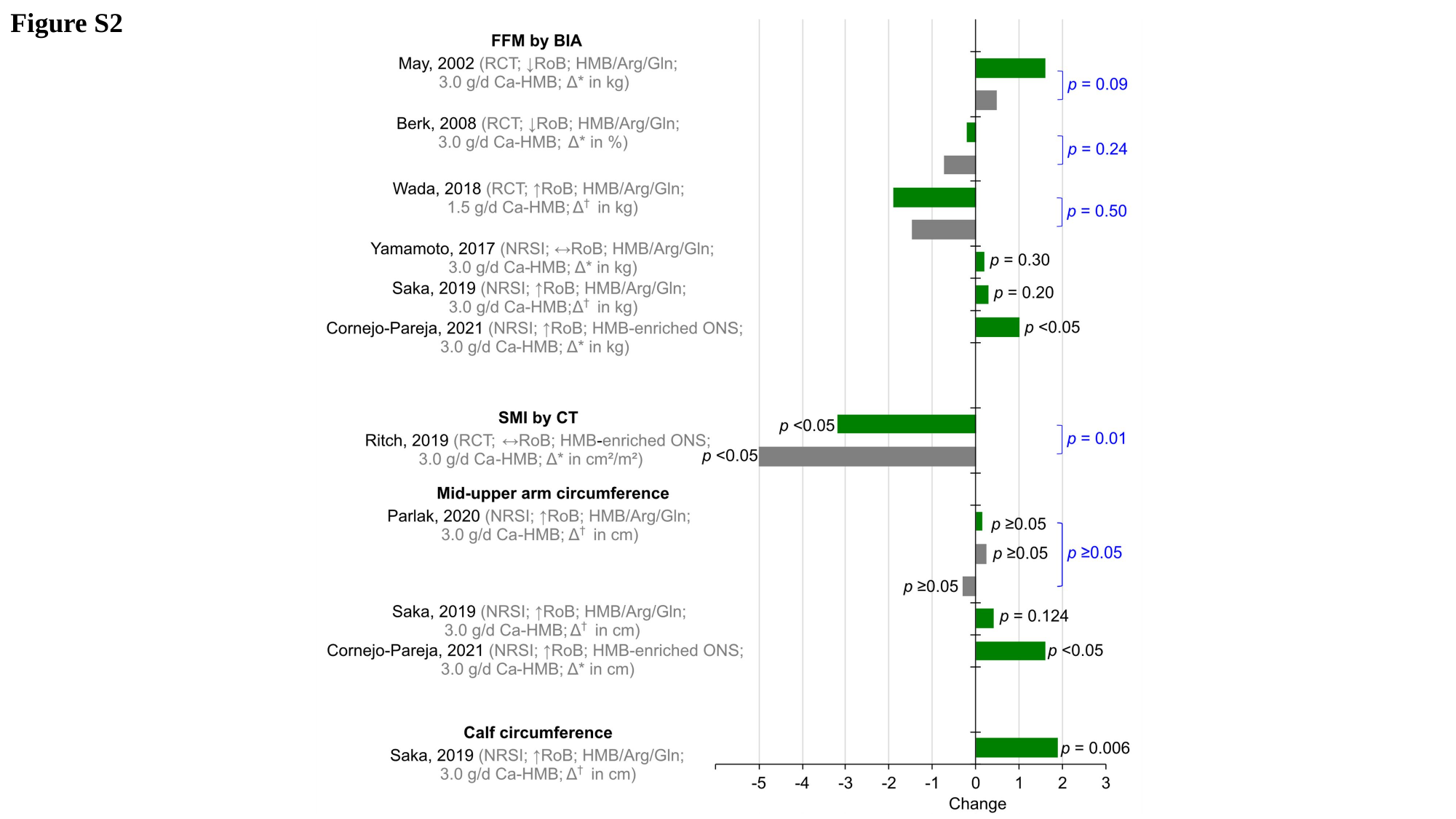

Figure S2

## Slide 4
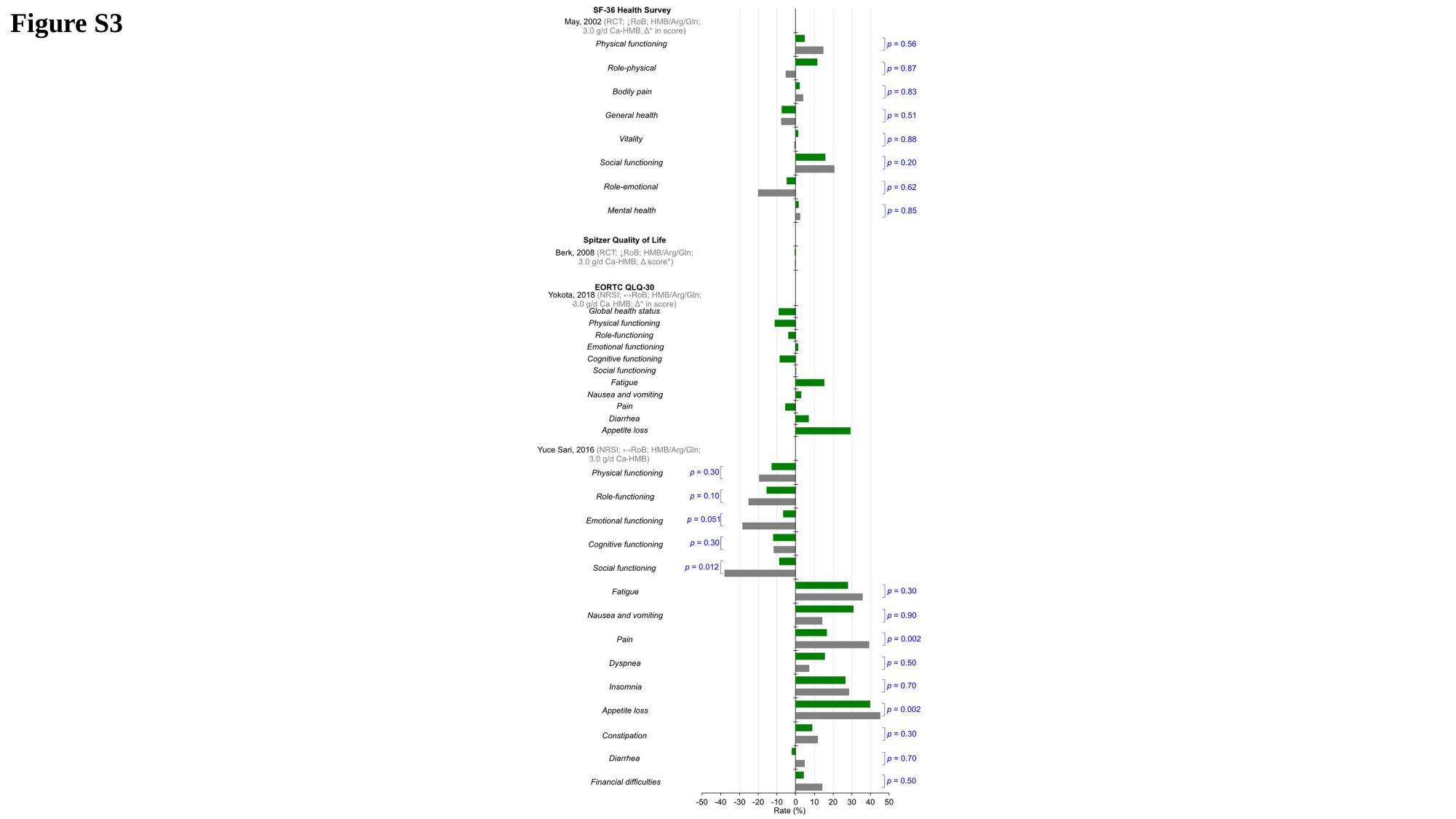

Figure S3

## Slide 5
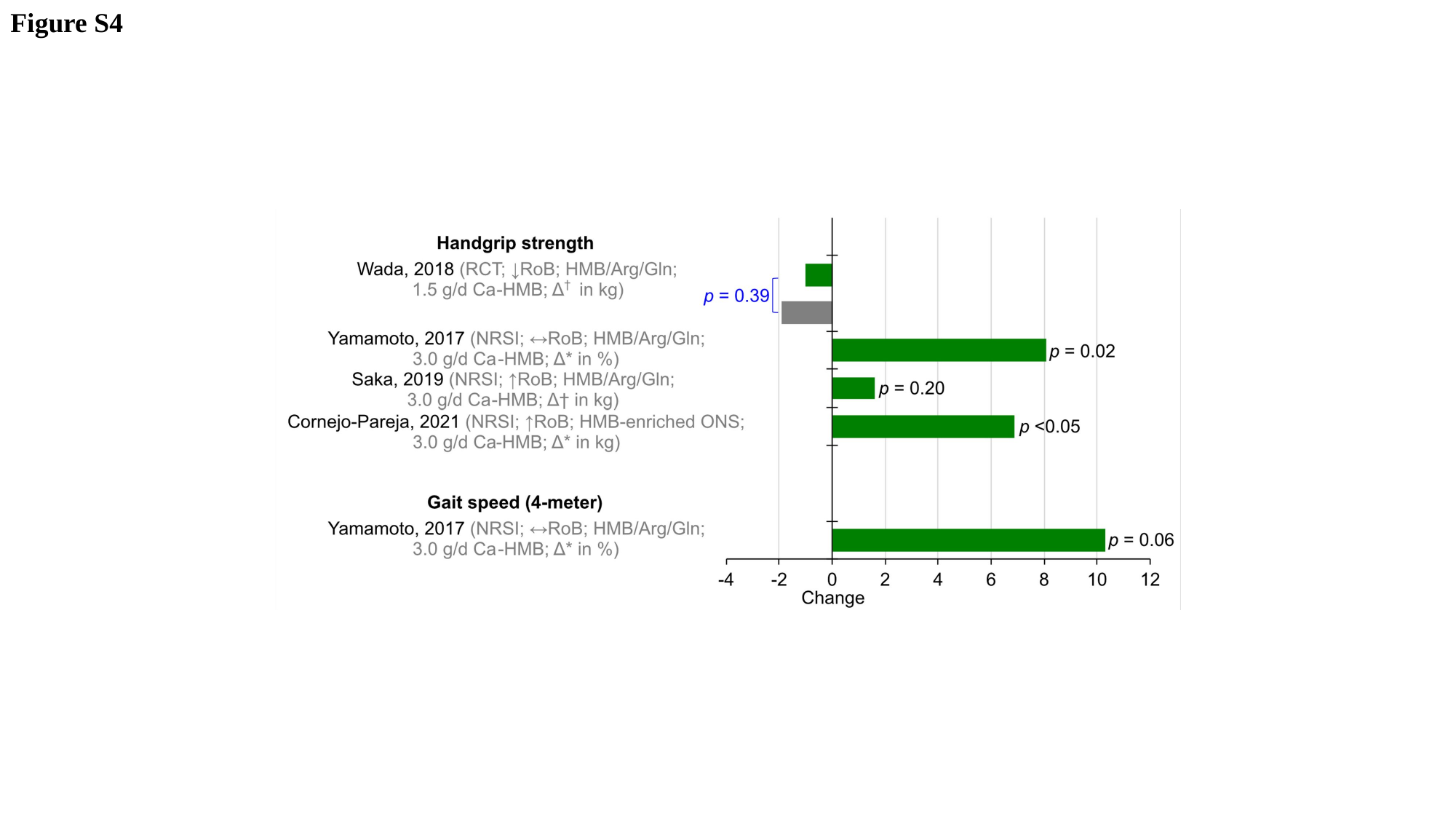

Figure S4

## Slide 6
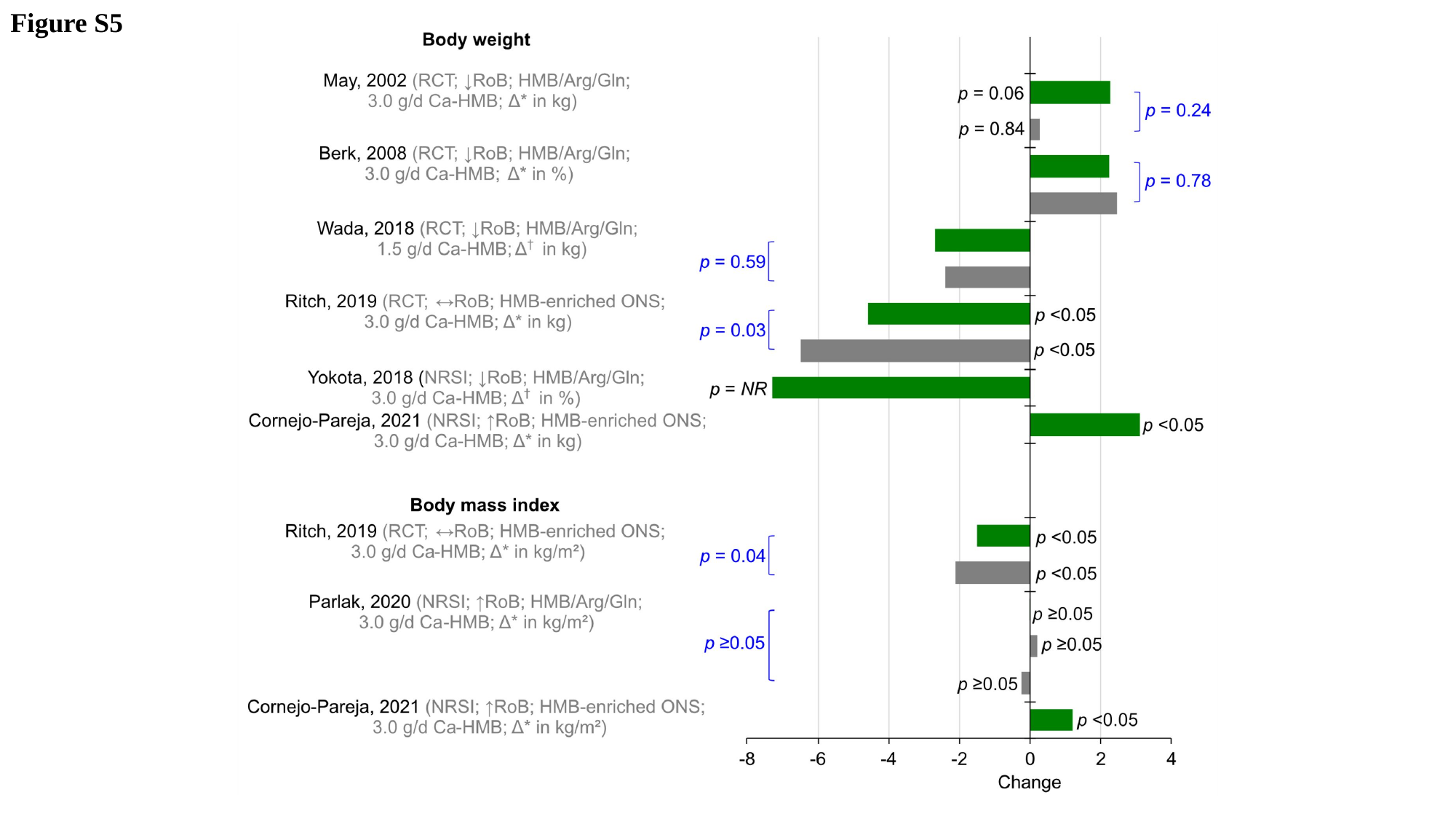

Figure S5

## Slide 7
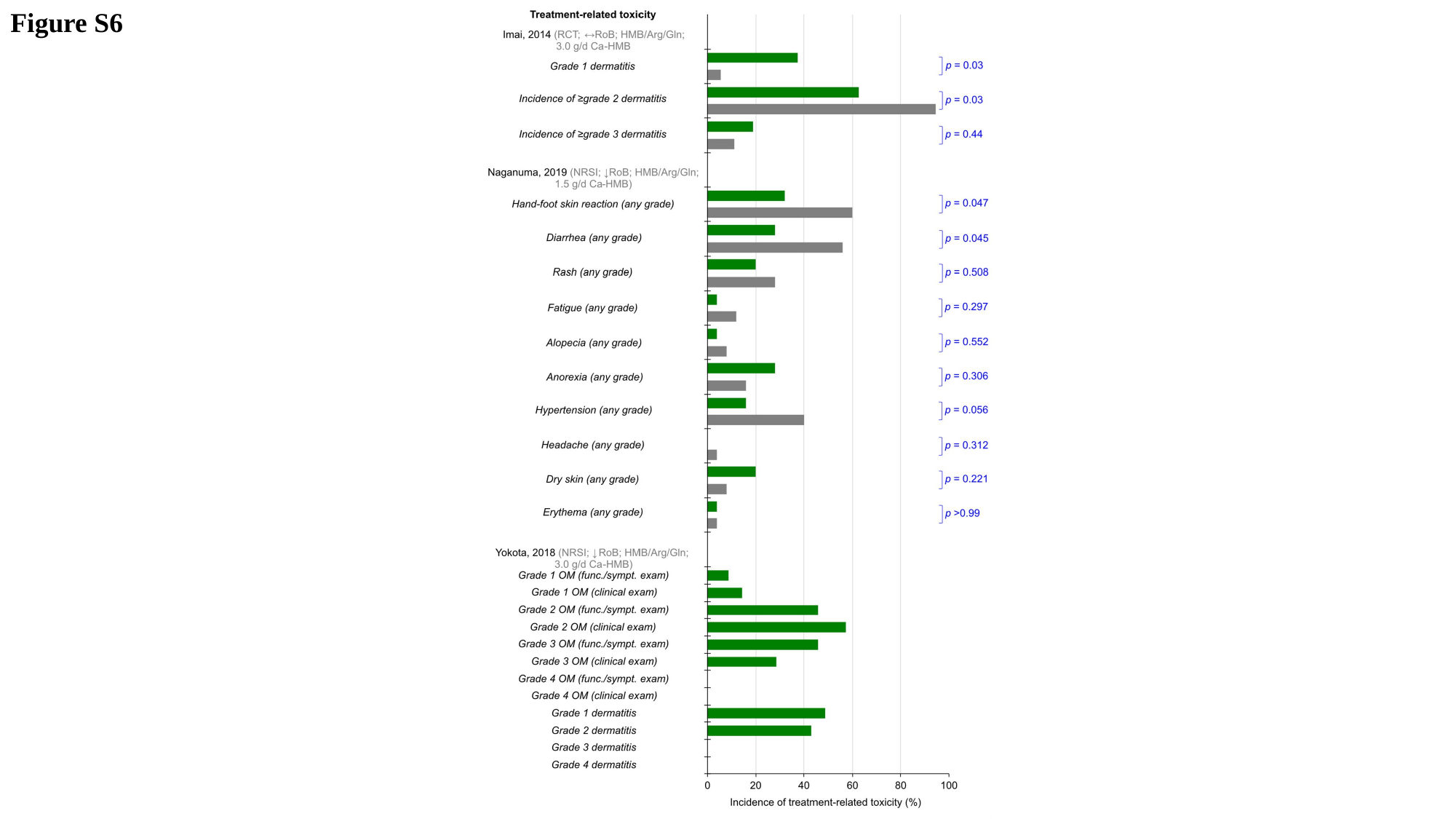

Figure S6

## Slide 8
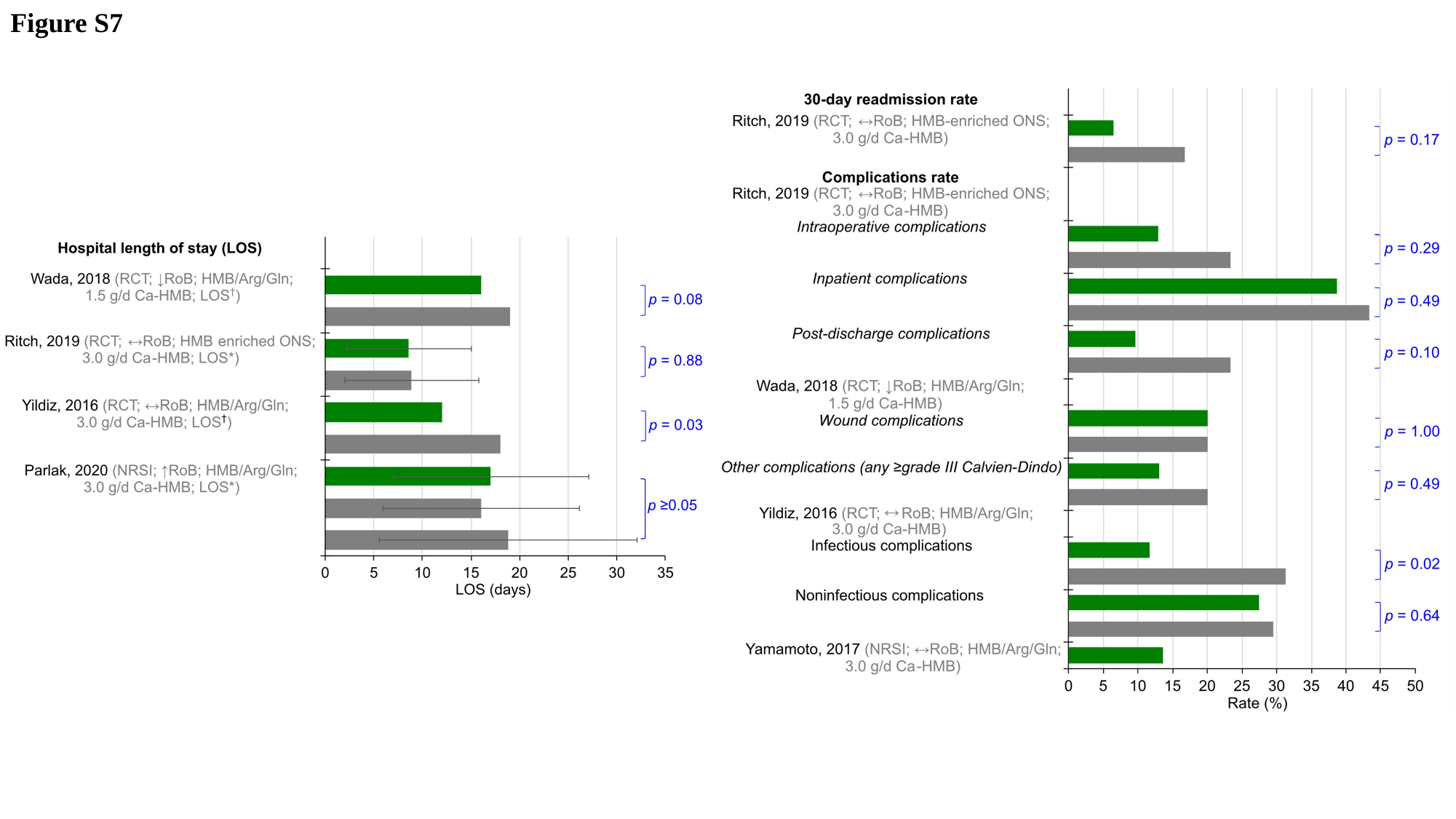

Figure S7

## Slide 9
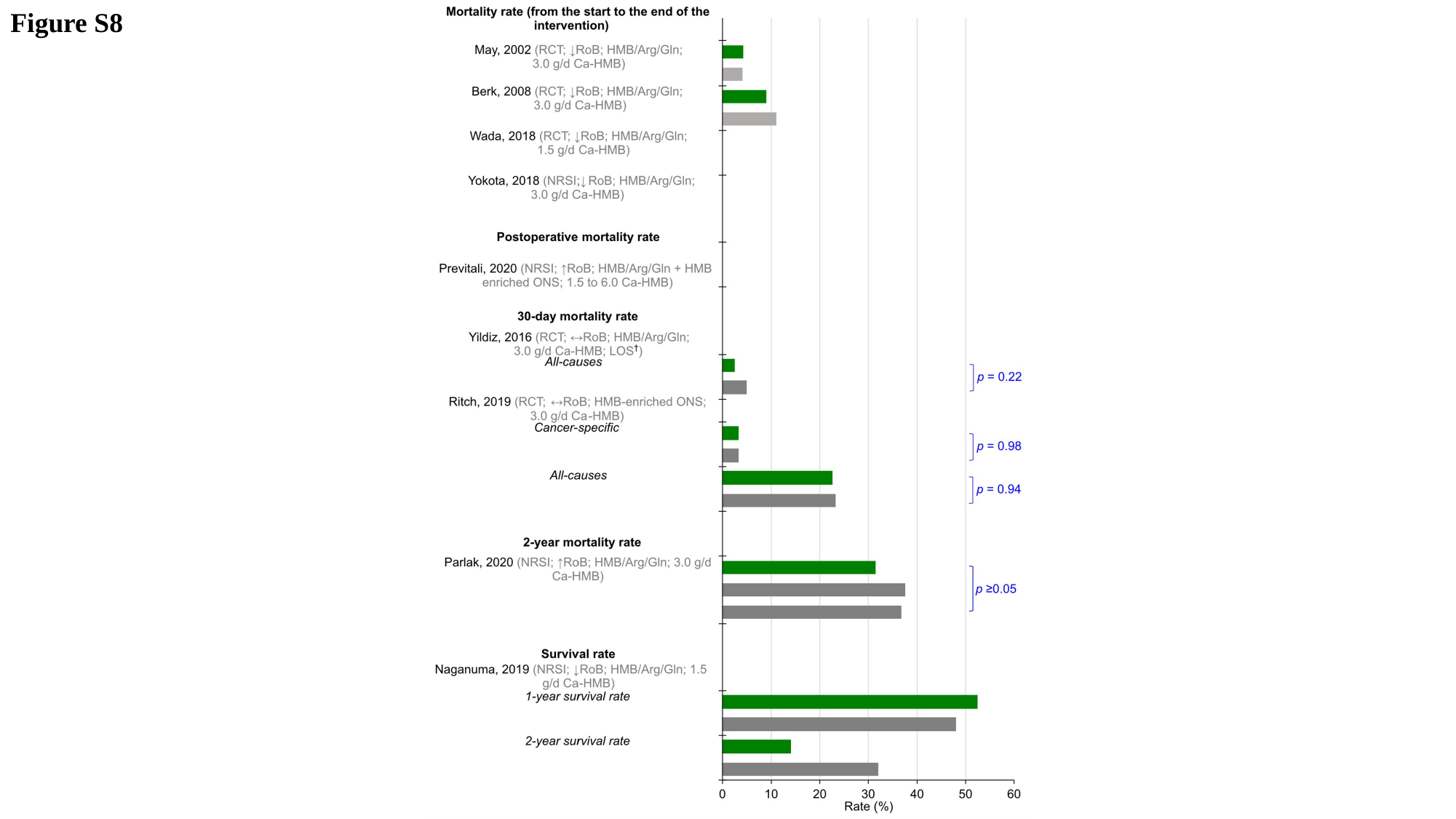

Figure S8

## Slide 10
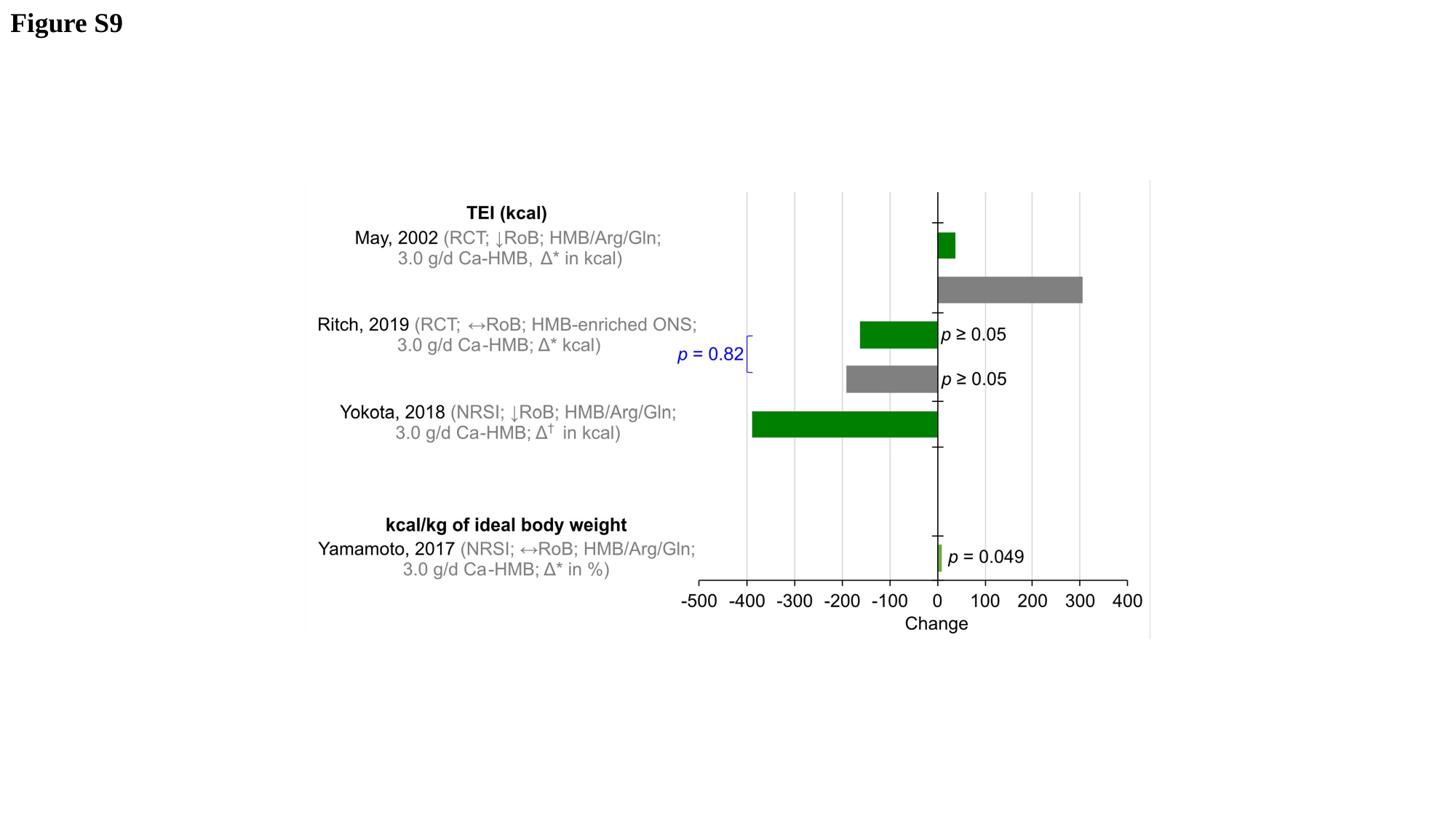

Figure S9

## Slide 11
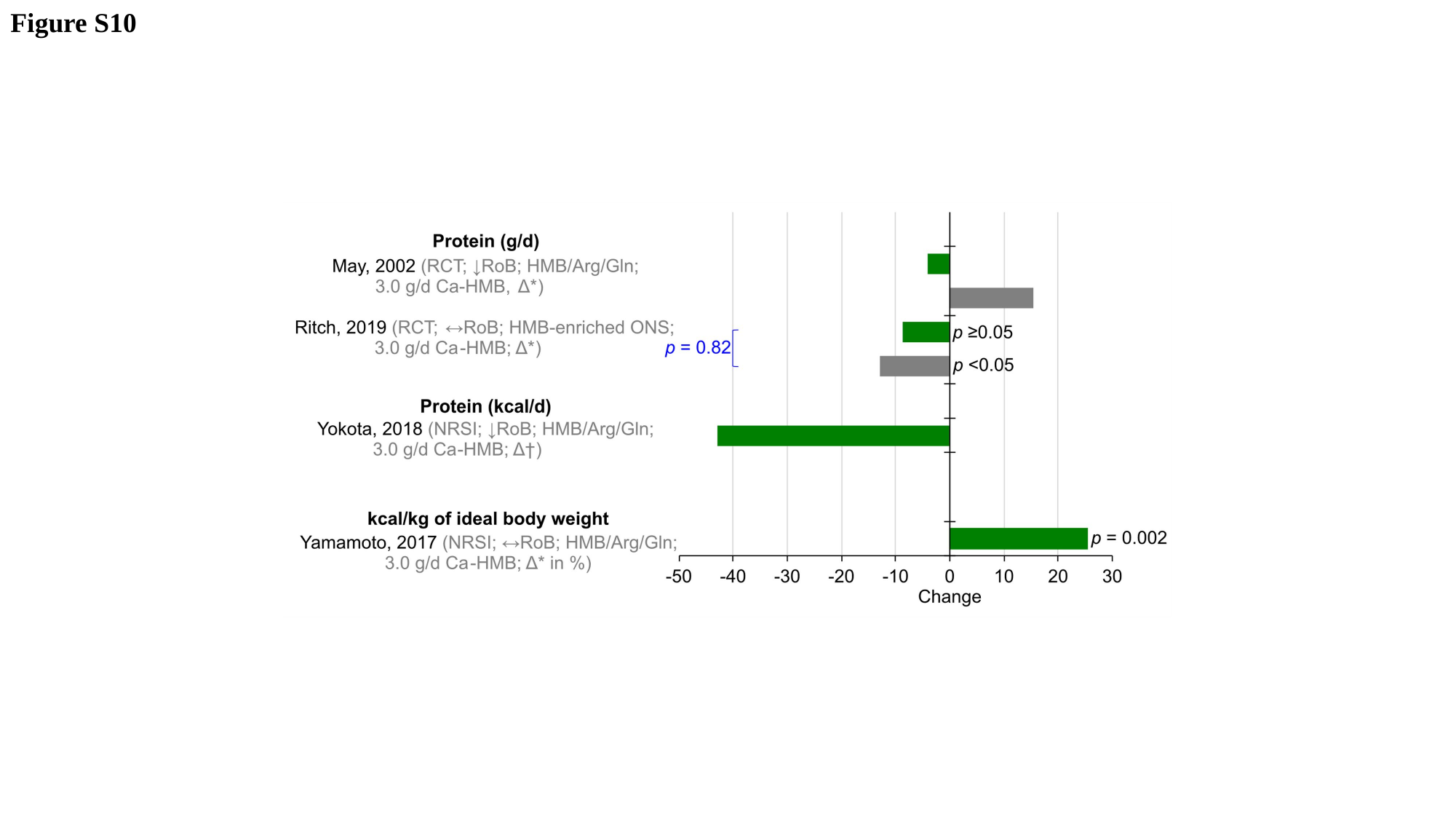

Figure S10

## Slide 12
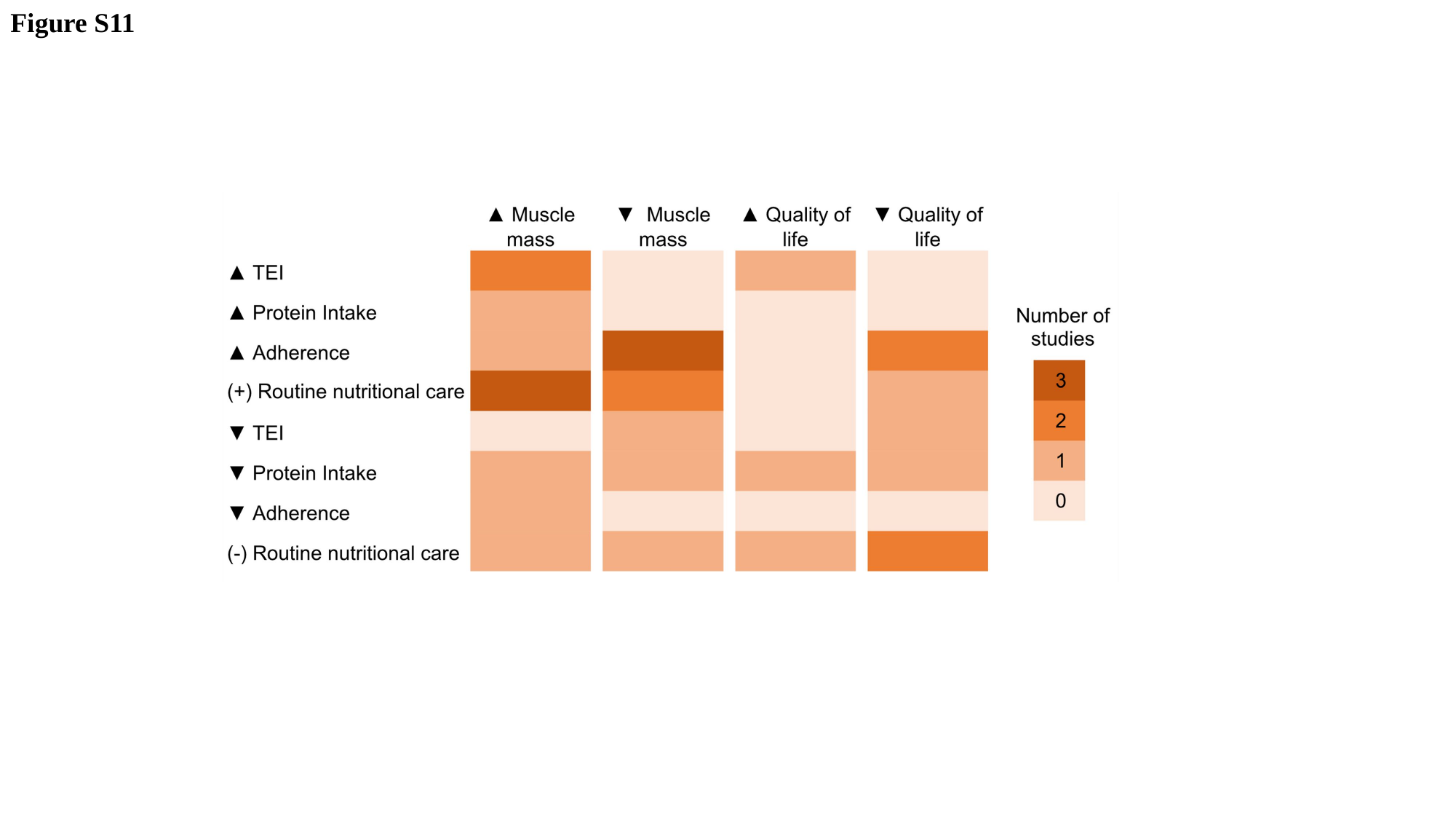

Figure S11

## Slide 13
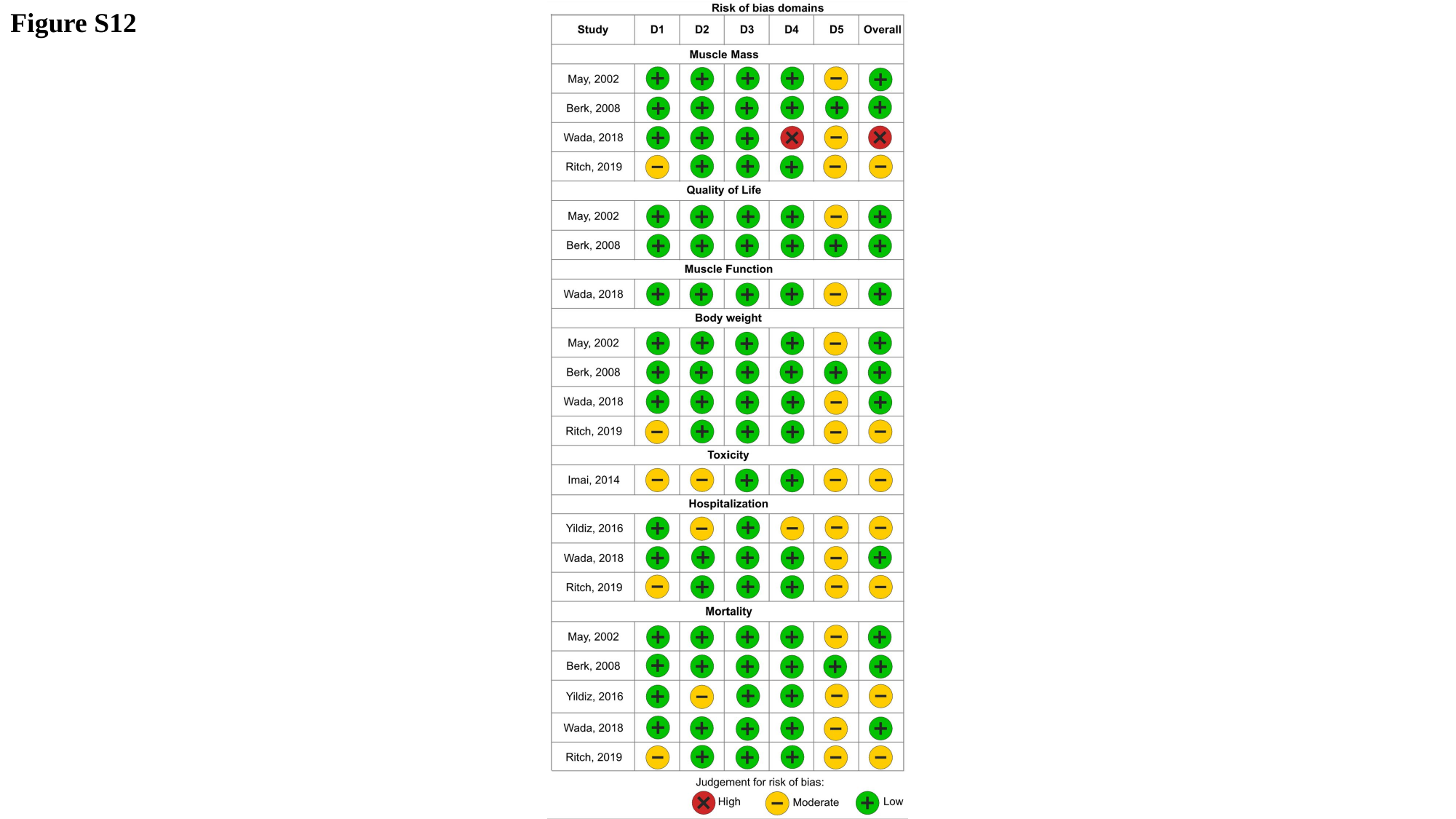

Figure S12

## Slide 14
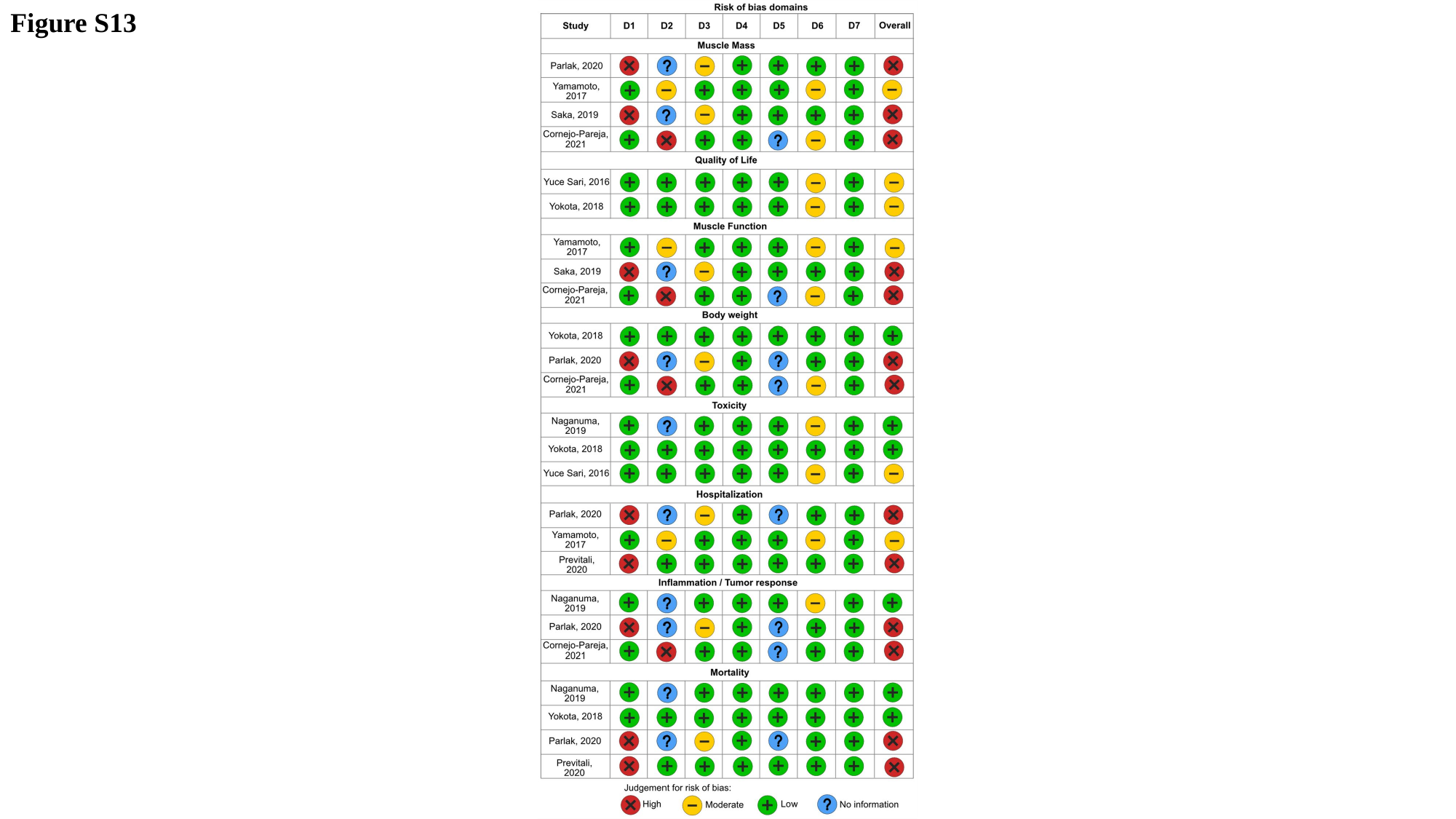

Figure S13
